# Reduction in chloroplastic ribulose-5-phosphate-3-epimerase decreases photosynthetic capacity in Arabidopsis

**DOI:** 10.3389/fpls.2022.813241

**Published:** 2022-10-14

**Authors:** Yonghong Li, Lianwei Peng, Xiaoqin Wang, Lin Zhang

**Affiliations:** ^1^ Beijing Advanced Innovation Center for Tree Breeding by Molecular Design, Beijing University of Agriculture, Beijing, China; ^2^ School of Biology and Brewing Engineering, TaiShan University, Taian, China; ^3^ Shanghai Key Laboratory of Plant Molecular Sciences, College of Life Sciences, Shanghai Normal University, Shanghai, China

**Keywords:** photosynthesis, Calvin-Benson cycle, carbon fixation, RPE, photosystem, ATP synthase

## Abstract

Chloroplast ribulose-5-phosphate-3-epimerase (RPE) is a critical enzyme involved in the Calvin-Benson cycle and oxidative pentose phosphate pathways in higher plants. Three Arabidopsis *rpe* mutants with reduced level of RPE were identified through their high NPQ (nonphotochemical quenching) phenotype upon illumination, and no significant difference of plant size was found between these *rpe* mutants and WT (wild type) plants under growth chamber conditions. A decrease in RPE expression to a certain extent leads to a decrease in CO_2_ fixation, *V*_cmax_ and *J*_max_. Photosynthetic linear electron transport was partially inhibited and activity of ATP synthase was also decreased in the *rpe* mutants, but the levels of thylakoid protein complexes and other Calvin-Benson cycle enzymes in *rpe* mutants were not affected. These results demonstrate that some degree of reduction in RPE expression decreases carbon fixation in chloroplasts, which in turn feedback inhibits photosynthetic electron transport and ATP synthase activity due to the photosynthetic control. Taken together, this work provides evidence that RPE plays an important role in the Calvin-Benson cycle and influences the photosynthetic capacity of chloroplasts.

## Introduction

Photosynthesis is one of the most influential processes for the earth biosphere. It generates O_2_ and converts CO_2_ into carbohydrates. Photosynthesis is basically composed of the light-dependent reactions and the carbon-fixing reactions (Johnson, 2016). In the light reactions, light excitation energy is absorbed and used to produce ATP and NAD(P)H for carbon fixation in the carbon-fixing reactions. The Calvin-Benson cycle (CBC) is the primary pathway of carbon fixation in the chloroplast stroma of higher plants, producing sugars and their derivatives for a series of pathways that are necessary for plant growth and development. During photosynthesis, the light and carbon-fixing reactions are tightly linked. When the activity of the carbon-fixing reactions is low, excess of ATP and NAD(P)H can inhibit photosynthetic electron transport and ATP synthase *via* the photosynthetic control mechanism (Foyer et al., 1990).

The Calvin-Benson cycle consists of 13 steps of chemical reactions catalyzed by 11 enzymes. Some intermediates and enzymes of CBC are shared with the respiratory carbon metabolic pathways, such as the oxidative pentose phosphate pathway (OPPP) and the glycolytic pathway (Kaiser, 1979; De Porcellinis et al., 2018). CBC is divided into three phases: (1) Carbon dioxide fixation: ribulose 1, 5-bisphosphate carboxylase/oxygenase (Rubisco) produces 3-phosphoglycerate (3-PGA) by carboxylation of ribulose 1, 5-bisphosphate (RuBP). This is the key step of the CBC (Bracher et al., 2017; Wilson and Hayer-Hartl, 2018). Rubisco is a multi-subunit complex with a molecular mass of ~550 kDa and contains eight large (RbcL) and eight small subunits (RbcS) (Bracher et al., 2017; Wilson and Hayer-Hartl, 2018). (2) Triose reduction: 3-PGA is reduced to glyceraldehyde 3-phosphate (G3P) by 3-phosphoglycerate kinase (PGK) and glyceraldehyde 3-phosphate dehydrogenase (GAPDH). (3) Pentose regeneration: triose phosphate isomerase (TPI), fructose 1,6-bisphosphate aldolase (FBA), fructose 1,6-bisphosphatase (FBPase), transketolase (TKL), ribulose-5-phosphate-3-epimerase (RPE), sedoheptulose-1,7-bisphosphatase (SBPase), ribose 5-phosphate isomerase (RPI), and phosphoribulokinase (PRK) are involved in the resynthesis of RuBP (Reyes-Prieto and Bhattacharya, 2007; Michelet et al., 2013; Sharkey, 2019). In addition to the CBC pathway, these enzymes usually have multiple homologs that have different locations in chloroplasts and cytoplasm, and they participate in other glucose metabolic pathways. For example, there are cytosolic and chloroplast PGK isozymes. The latter have been hypothesized to originate from a cyanobacterial ancestor (Massange-Sanchez et al., 2020). There are also four different GAPDH isoforms localized in the cytosol or in chloroplasts of which only GAPA/B is involved in the CBC (Gani et al., 2016). FBPases are homotetrameric enzymes with different isoforms cFBP (in chloroplasts) and cyFBP (in cytosol) in plants (Serrato et al., 2018).

To enhance CO_2_ fixation and photosynthesis, some CBC enzymes were found to be overexpressed in various oxygenic phototrophs. However, their effects on photosynthetic productivity and growth are dependent on the particular enzyme, species, and other factors (De Porcellinis et al., 2018). Other enzymes are thought to have a greater control over carbon flow during photosynthesis than Rubisco, such as SBPase, FBA, and TKL (Raines, 2003; Mu et al., 2021). Overexpression of SBPase and FBPase has demonstrated the potential of increase carbon fixation, photosynthesis to increase growth, biomass and even seed yield in transgenic plants (Lefebvre et al., 2005; Tamoi et al., 2006; Rosenthal et al., 2011; Simkin et al., 2017; López-Calcagno et al., 2020). Overexpression of TKL gene promotes chilling tolerance by increasing the activities of photosynthetic enzymes in cucumber plants (Bi et al., 2019), However, overexpression of plastid transketolase in tobacco results in a thiamine auxotrophic phenotype, which may contribute to the complex regulatory mechanisms maintaining thiamine homeostasis in plants (Khozaei et al., 2015). The importance of the activities of individual enzymes in the CBC cycle for carbon fixation and photosynthesis is complex and not well understood.

RPE catalyzes the interconversion of ribulose-5-phosphate and xylulose-5-phosphate in the CBC and OPPP (Favery et al., 1998). In the Arabidopsis genome, only *At5g61410* was designated as RPE and located in plastids. The Arabidopsis chloroplast *RPE* deletion mutant *rpe* can be germinated only when exogenous carbohydrates are added and are dwarf-like with light-green leaves (Favery et al., 1998; Xiong et al., 2009). It was found that the *RPE* gene affects the early stage of giant cell formation induced by the root-knot nematode (Favery et al., 1998). The lethality of the homozygous *rpe* mutant may be mainly due to the adverse effect on the production of Ru5P in the OPPP pathway, resulting in reduced purine synthesis required for embryonic development (Andriotis and Smith, 2019). In addition to RPE, two other proteins encoded by *At1g63290* and *At3g01850* were proposed to be cytosolic RPE (cyRPE) (Kruger and von Schaewen, 2003; Baune et al., 2020). Cytosolic RPEs have also been shown to be involved in the OPPP which is critical for production of NADPH (Kopriva et al., 2000).

In this study, we have isolated three *rpe* mutants which accumulate low levels of chloroplast RPE protein in Arabidopsis. Reduced CO_2_ fixation and photosynthetic electron transport was found in these *rpe* mutants under high light conditions suggesting that RPE plays an important role in the Calvin cycle and influences the photosynthetic capacity of chloroplasts.

## Materials and methods

### Plant materials and growth conditions

The mutant *rpe-1* was isolated from the pSKI015 T-DNA insertion Arabidopsis mutant pool according to the high level of NPQ upon illumination. T-DNA insertion mutants *rpe-2* (SAIL_1271_E12) and *rpe-3* (SALK_023919C) were obtained from NASC (Nottingham Arabidopsis Stock Centre). Wild-type *Arabidopsis thaliana* (Col ecotype) and mutants were grown on soil under greenhouse conditions (16 h/8 h: light/dark photoperiod, 50 μmol photons m^-2^ s^-1^, 23°C).

### Chlorophyll fluorescence measurements

Imaging of chlorophyll fluorescence was performed according to Zhang et al. (2016) with an IMAGING-PAM fluorometer (Walz, Effeltrich, Germany). NPQ induction curves were measured with default programs also using IMAGING-PAM fluorometer and leaves were selected to derive NPQ data.

Light-intensity dependence parameters of ETR II, NPQ, and 1-qL were measured after illumination for two minutes using a Dual-PAM-100 (Walz) with a series of light intensities (9, 38, 77, 117, 221, 397, 665, 1031 μmol photons m^-2^ s^-1^) as previously reported (Zhang et al., 2016). Light intensity-dependent ETR I and oxidation of the donor side of PSI was measured with a Dual-PAM-100 under a series of light intensities (22, 68, 143, 271, 476, 756, 1194, 1865 μmol photons m^-2^ s^-1^) after turning on light for 20 s and calculated automatically using Dual-PAM-100 software (Zhang et al., 2016).

### Protein extraction and protein gel blot analyses

Thylakoid membrane and stromal proteins isolation, immunoblotting, BN-PAGE and 2D-PAGE analysis were performed as previously described (Zhang et al., 2018). Thylakoid membrane protein was determined by the acetone method for chlorophyll content, chloroplast stromal proteins were determined by dye-binding method according to Bradford.

Antibodies against D1 (ATCG00020, PHY0057), D2 (ATCG00270, PHY0060), LHCII (AT1G76570, PHY0471S), PsaA (ATCG00350, PHY0342), CF1α/β (ATCG00480, PHY0312), PetC (AT4G03280, PHY2293S), NDHH (ATCG01110, PHY2018A), PGRL1 (AT4G22890, PHY0234A), RbcL (ATCG00490, PHY0346), RPE (AT5G61410, PHY0616), FBA2 (AT4G38970, PHY0406S), PGK1 (AT3G12780, PHY0405S), PRK (AT1G32060, PHY0100A), RCA (AT2G39730, PHY0400S), RPI (AT3G04790, PHY0402S), TPI (AT2G21170, PHY0409S), TKL1 (AT3G60750, PHY0407S), GAPA1 (AT3G26650, PHY0408S), SBPase (AT3G55800, PHY0410S) were obtained from PhytoAB (USA).

### Subcellular localization of GFP fusion proteins

Subcellular localization was performed as described by Zhang et al. (2016) and full-length of *RPE* cDNA was cloned into pBI221 vector to express the RPE-GFP fusion protein.

### ATP synthase activity measurement

The activity of ATP synthase was measured as described previously (Zhang et al., 2018) using a Dual-PAM-100 (Walz) with a P515/535 emitter-detector module except that leaves were illuminated for 10 min with 167, 325, and 606 μmol photons m^-2^ s^-1^ red light after overnight dark-adaption.

### Gas exchange measurements

Photosynthetic gas exchange was measured using a GFS-3000 gas exchange measuring system (Walz). Air temperature in the leaf chamber and the relative humidity were maintained at 23°C and 50%, respectively. Air concentrations were controlled at 400 ppm CO_2_ and 21% O_2_. The CO_2_ assimilation rate was determined at 103, 197, and 756 μmol photons m^-2^ s^-1^ red light with the leaf of mutant and wild-type plants grown for 4 weeks. The steady-state of gas exchange was recorded after 10 min illumination.

### *A/Ci* photosynthetic gas exchange measurements

The *A/Ci* curve was measured using the GFS-3000 gas exchange system (Walz, Effeltrich, Germany) with leaves of 6-week-old plant. For each line, the measurement was performed on at least five individual plants at 25°C, relative humidity of 60% and 400 ppm of CO_2_. Leaves were illuminated using a red light source of Dual-PAM-100 (Walz, Effeltrich, Germany) attached to the gas-exchange system at 1000 μmol photons m^-2^ s^-1^. Net photosynthesis (*A*) was measured with three replications under a series of CO_2_ concentrations as following: 400, 50, 100, 150, 200, 300, 400, 500, 700, 1000, 1500, and 2000 ppm. *A/Ci* curves were analyzed using the equations of Farquhar et al. (1980). The maximum Rubisco carboxylation rate (*V*_cmax_) and RuBP regeneration rate (*J*_max_) were estimated according to the *A/Ci* curves using the method described previously (Dubois et al., 2007).

## Results

### Isolation of the *rpe-1* mutant

**Figure 1:**
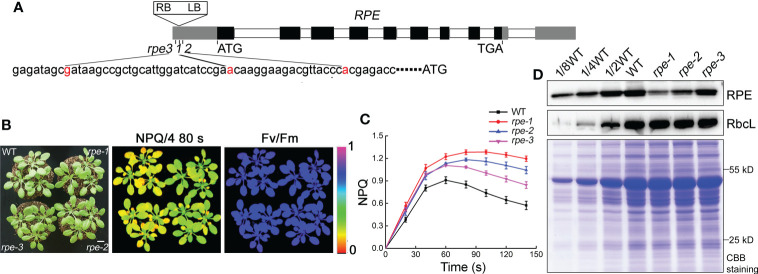
Characterization of the *rpe* mutants. **(A)** Schematic representation of *RPE*. The positions of the T-DNA insertions in the three *rpe* mutants are shown with red nucleotides. **(B)** Growth, NPQ, and Fv/Fm phenotypes of WT and *rpe* mutants. Four-week-old plants grown on soil at 50 μmol photons m^-2^ s^-1^ (left panels). Signal intensities for NPQ and Fv/Fm are indicated according to the color scale (0 to 1.0) on the right. The bar represents one centimeter. **(C)** NPQ induction curves of the *rpe* mutants and WT. NPQ was calculated as (Fm-Fm’)/Fm’. Data are presented as the means ± SD (n=4). **(D)** Immunoblot analysis of RPE protein in the *rpe* mutants. Chloroplast stroma was isolated and immunoblot analyses were performed. RbcL was used as loading control. Equal amounts of chloroplast stromal proteins were loaded on the gels. The gels were stained with CBB for visualization of the proteins.

The mutant *rpe-1* was isolated from a pSKI015 T-DNA insertion mutant pool of Arabidopsis through the high level of NPQ (non-photochemical quenching) after illumination (**Figures 1B, C**; **Supplementary Figure 1**). The *rpe-1* mutant did not exhibit a visible growth phenotype under growth chamber conditions, such as leaf size and chlorophyll content (**Supplementary Figure 2**). The maximum photochemical efficiency (Fv/Fm) was comparable to WT (**Figure 1B**; **Supplementary Figure 1** **)**, indicating that the function of Photosystem II (PSII) is not affected in the mutant. In WT plants, NPQ is induced to approximately 0.9 within 60 s of illumination with AL (Actinic light). Then NPQ was relaxed within two min due to the activation of ATP synthase and the CBC reactions in the chloroplast stroma (**Figure 1C**, Zhang et al., 2016). In contrast, the NPQ level in *rpe-1* increased continuously to approximately 1.2 after 80 s of illumination and did barely relax during the following period of illumination (**Figure 1C**).

### Identification of the *rpe* mutations

Thermal asymmetric interlaced polymerase chain reaction (TAIL-PCR) revealed that the T-DNA was inserted in the 5-UTR of the chloroplast *RPE* gene (*At5g61410*) in the *rpe-1* mutant (**Figure 1A**). We obtained two other mutants *rpe-2* and *rpe-3* in which the T-DNA was also inserted in the 5-UTR of *RPE* (**Figure 1A**). All three alleles had similar high NPQ phenotypes upon illumination, although the degree of elevation was different (**Figures 1B, C**). These results indicate that the T-DNA insertion in the 5-UTR of *RPE* is responsible for the high-NPQ phenotype in the *rpe* mutants.

**Figure 2:**
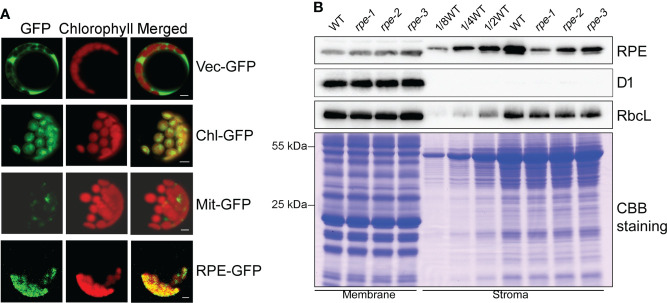
Subcellular localization of RPE. **(A)** Subcellular localization of RPE by GFP assay. RPE-GFP, RPE-GFP fusion; Vec-GFP, empty plasmid control; Chl-GFP, chloroplast control; Mit-GFP, mitochondrial control. Bars=5 μm. **(B)** Immunolocalization analysis of RPE. Intact chloroplasts isolated from WT and the *rpe* mutants were fractionated into membrane and stromal fractions, and immunoblot analyses were performed. D1 and RbcL were used as loading and fractionation controls. Thylakoids membrane protein containing equal amounts of chlorophyll and equal amounts of chloroplast stromal proteins were loaded on the gels. The gels were also stained with CBB for visualization of the proteins.

RPE is involved in the CBC and OPPP. Transient expression of RPE-GFP fusion protein in Arabidopsis protoplasts confirmed that RPE is specifically localized in chloroplasts (**Figure 2A**). Immunoblot analyses of chloroplast membrane and stromal proteins showed that RPE is present in the chloroplast stroma (**Figure 2B**) consistent with the localization of the CBC reactions. As expected, RPE protein is reduced to less than 50% of WT level in the three *rpe* mutants (**Figures 1D**, **2B**). Although the T-DNA were all inserted into the 5-UTR in *rpe*s, their germplasm sources were not consistent, resulting in inconsistent protein expression levels. The accumulation of RPE in *rpe-1* is less than in *rpe-2* and *rpe-3*, and reduced to ∼20% of wild-type levels (**Figures 1D**, **2B**) which is consistent with the NPQ induction curves of these three mutants (**Figures 1B, C**).

### The abundance of the major thylakoid protein complexes did not change in the *rpe* mutants

**Figure 3:**
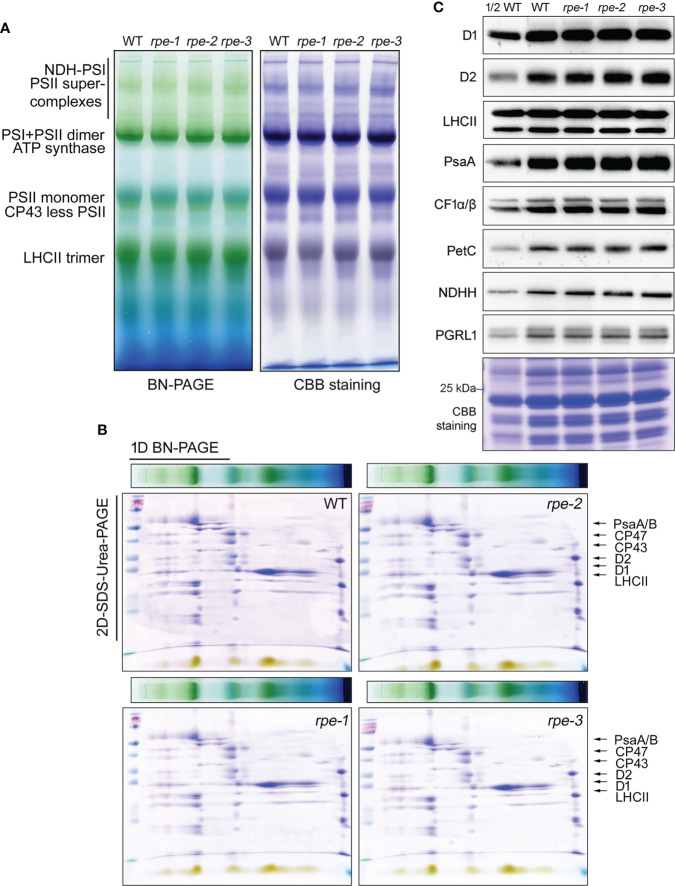
Analysis of the photosystem complexes and their core proteins in WT and the *rpe* mutants. **(A, B)** BN-PAGE **(B)** and 2D SDS-Urea-PAGE **(C)** analysis of the thylakoid protein complexes. Thermo Scientific PageRuler Prestained Protein Ladder is used for the protein molecular weight marker (Thermo, 26616). **(C)** Immunoblot analysis of representative thylakoid proteins. Thylakoid membranes isolated from the WT and the *rpe* mutants were separated by 15% SDS-Urea-PAGE and then probed with antibodies against individual subunits of PSII (D1, D2 and LHCII), PSI (PsaA), chloroplast ATP synthase (CF_1_α/β), Cyt *b_6_f* (PetC), NADH dehydrogenase-like complex (NDHH), and PGR5/PGRL1 complexes (PGRL1). Thylakoid proteins containing equal amounts of chlorophyll were loaded.

By using NPQ screening system, we have successfully isolated three mutants accumulating low amounts of chloroplast ATP synthase (*bfa1*, *bfa2*, and *bfa3*, Zhang et al., 2016; Zhang et al., 2018; Zhang et al., 2019). To investigate whether the *rpe* mutants have similar phenotypes of those three mutants, thylakoid protein complexes were analyzed by blue-native PAGE and immunoblot analyses. As shown in **Figure 3**, thylakoid protein complexes of PSI-NDH supercomplex, PSI monomer, PSII supercomplexes, PSII dimer, PSII monomer, CP43-less PSII, trimeric LHCII, and chloroplast ATP synthase were readily detected in the WT and the *rpe* mutants by BN-PAGE (**Figure 3A**). The BN-gel was further subjected to 2D SDS-urea-PAGE to resolve into individual subunits. No obvious difference was found in the levels of these subunits between WT and the *rpe* mutants (**Figure 3B**; **Supplementary Figure 3**). The CF_1_α/β/γ subunits were detected at the position of the intact ATP synthase and the CF_1_ subcomplex at similar levels in WT and the *rpe* mutants indicating that the amount of ATP synthase is not reduced in the mutant. This conclusion is further supported by the immunoblot analysis using a series of antibodies against some of the major subunits of the photosynthetic complexes such as photosystem II (D1, D2 and LHCII), PSI (PsaA), cytochrome *b6f* complex (PetC) and ATP synthase (CF_1_α/β). The levels of these subunits were identical between the *rpe* mutants and the WT (**Figure 3C**). The level of NADH dehydrogenase-like complex (NDHH) and PGR5/PGRL1 complex (PGRL1) is also not affected in the *rpe* mutants. These results indicate that the *RPE* deficiency does not affect the accumulation of the major thylakoid complexes involved in photosynthetic electron transport.

### CO_2_ fixation is decreased in the *rpe* mutants

**Figure 4:**
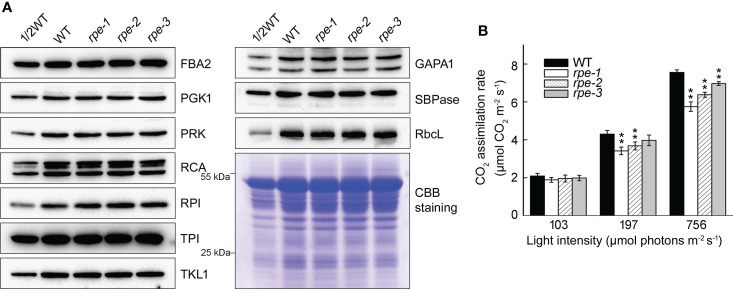
Characterization of Calvin-Benson cycle from WT and the *rpe* mutants. **(A)** Immunoblot analysis of Calvin-Benson cycle enzymes in WT and the *rpe* mutants. Chloroplast stroma was isolated and immunoblot analyses were performed using the indicated antibodies. Equal amounts of chloroplast stromal proteins were loaded. The gels were stained with CBB for visualization of the proteins. **(B)** CO_2_ assimilation rate of WT and the *rpe* mutants under different light intensities. Values are means ± SD (n=3). Significant differences were performed using the Student’s t-test (**P < 0.01).

To determine whether the amounts of other CBC enzymes change when the level of the RPE protein is reduced, we determined their accumulation by immunoblotting. We found that the level of 10 plastid-localized CBC enzymes was almost identical between WT and the *rpe* mutants (**Figure 4A**) suggesting that the reduction of RPE does not impact the level of other CBC enzymes. Expression of CBC enzymes may not be tightly co-regulated. To explore the impact of *RPE* deficiency, the CO_2_ assimilation rate was measured under different light intensities and atmospheric CO_2_ levels. Before the analysis, seedlings were incubated in the dark for 2 h to completely inactivate the CBC enzymes. Under low light conditions at 103 μmol photons m^-2^ s^-1^, the CO_2_ assimilation rate of the *rpe* mutants was comparable to WT (**Figure 4B**). This result is consistent with the similar plant size of WT and the *rpe* mutants grown in chamber conditions. Under 197 μmol photons m^-2^ s^-1^, the CO_2_ assimilation rate of *rpe-1 and rpe-2* was slightly reduced compared to WT (**Figure 4B**). Under 756 μmol photons m^-2^ s^-1^, even the CO_2_ assimilation rate of *rpe-3* was slightly reduced compared to WT. Although this reduction was more pronounced under 756 μmol photons m^-2^ s^-1^, the CO_2_ assimilation rate was reduced by only 25% in *rpe-1* (**Figure 4B**). This indicates that, in the *rpe* mutants, about 20% of RPE protein is capable to assimilate 75% of CO_2_ compared to WT, at least under our experimental conditions (**Figures 4B**, **1D**).

**Figure 5:**
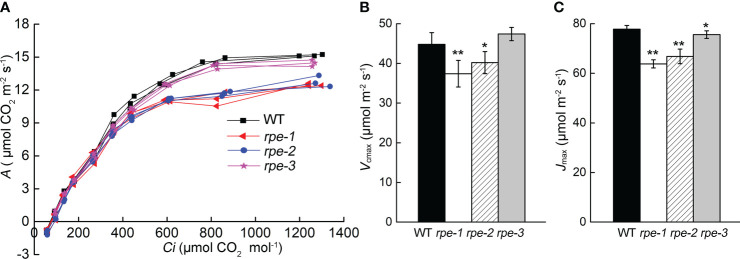
CO_2_ assimilation rate **(A)** to changes in intercellular CO_2_ (*Ci*). **(A)**The curve of *A* to *Ci* (the *A/Ci* curve). Individual measurements on three plants were presented. **(B, C)** *V*_cmax_ **(B)** and *J*_max_ **(C)** of the response of *A* to *Ci*. Values are means ± SD (n=5) (* P < 0.05; **P < 0.01).

Further analysis of the photosynthetic rates of the *rpe* mutants and WT was carried out by determining the response of CO_2_ assimilation (*A*) to changes in intercellular CO_2_ concentration (*Ci*). The *A/Ci* curve represents the carbon assimilation in mesophyll cells for a given carbon supply through stomata. The dependence of *A/Ci* is determined by the degree of Rubisco saturation, involving carboxylation, regeneration, or the export of TPU (triose phosphate utilization), referred to as “Rubisco limited” or “RuBP limited”, and “TPU limited”. Maximum carboxylation rate (*V*_cmax_) and RuBP regeneration rate (*J*_max_) are valuable metrics of photosynthetic performance, which was estimated by FvCB model (Farquhar et al., 1980; Dubois et al., 2007). The *rpe-1* and *rpe-2* had a significantly different response of *A* to that of WT at high *Ci* (**Figure 5A**). The maximum Rubisco carboxylation rate (*V*_cmax_) and RuBP regeneration rate (*J*_max_) in two mutants also showed a decrease relative to the wild-type (**Figures 5B, C**). The *V*_cmax_ of the *rpe-*3 mutant with the least decrease in RPE protein was not different from those of WT, but *J*_max_ showed a slight decrease (**Figures 5B, C** **)**.

### Photosynthetic electron transport and ATP synthase activity are partially inhibited in the *rpe* mutants

**Figure 6:**
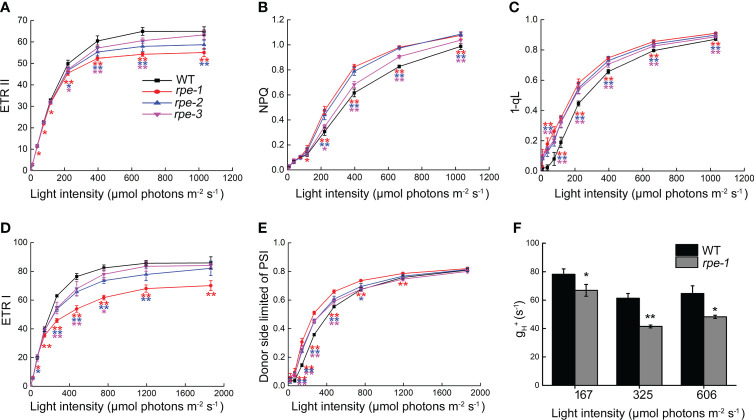
Analysis of light intensity dependence of photosynthetic parameters of WT and the *rpe* mutants. **(A)** Electron transport rate around PSII (ETR II). **(B)** Nonphotochemical quenching (NPQ). **(C)** 1-qL of PSII. qL means the fraction of open PSII centers, and 1-qL reflects the PQ redox state of PSII. **(D)** Electron transport rate around PSI (ETR I). **(E)** Donor-side limited PSI (with donor-side oxidized). **(F)** Analysis of ATP synthase activity in WT and *rpe-1*. Values are means ± SD (n=3-6).Significant differences between WT and the *rpe* mutants are indicated by asterisk according to the Student’s t test (*P < 0.05; **P < 0.01), The red, blue, and pink asterisk represents significant differences between WT and *rpe-1, rpe-2*, *rpe-3*, respectively.

In order to determine possible effects on photosynthetic electron transport in the *rpe* mutants, we analyzed the chlorophyll fluorescence parameters under different light intensities. Electron transport rates through PSII (ETR II) and through PSI (ETR I) are indicators of the relative of electron flow through PSII and PSI. Compared with WT, lower ETR II and ETR I values were observed in the *rpe* mutants, especially the *rpe-1* mutant with the lower RPE expression decreased more obviously at light intensities greater than 100 μmol photons m^-2^ s^-1^ (**Figures 6A, D** **)**. The steady-state NPQ in the *rpe* mutants was higher compared with WT at light intensities greater than 100 μmol photons m^-2^ s^-1^(**Figure 6B**) suggesting that more protons accumulated in the thylakoid lumen in the *rpe* mutants compared with WT. The increase of steady-state NPQ in the rpe mutants was also consistent with the decrease of RPE expression. We also analyzed the photosynthetic parameter 1-qL, which shows the degree of the reduction state of the plastoquinone pool, in which it is assumed that the redox level of the primary quinone acceptor (Q_A_) in PSII is in rapid equilibrium with the redox level of the plastoquinone pool (Ohnishi et al., 2021). Higher 1-qL were observed in the *rpe* mutants (**Figure 6C**) suggesting that the redox state of plastoquinone on the PSII acceptor side is reduced in the *rpe* mutants than in WT. Furthermore, the PSI donor side was more oxidized than WT levels (**Figure 6E**). All these results indicate that photosynthetic electron transport is affected in the *rpe* mutants and limitation occurs between PSII and PSI.

To detect the changes of ATP synthase activity, we analyzed the light-intensity dependence of gH^+^ which reveals the conductivity of the thylakoids to protons in *rpe-1* (**Figure 6F**). gH^+^ was reduced to 67%-85% of wild-type levels in *rpe-1* at the light intensities investigated certifying that ATP synthase activity is reduced in *rpe-1* (**Figure 6F**).

## Discussion

Some of the enzymes involved in the plastid OPPP and CBC pathways are critical for embryo development in *Arabidopsis*. Their absence usually leads to seed abortion, non-viable seeds, or non-viable seedlings (Bryant et al., 2011). Mutations in enzymes (such as PGL3, PGD, and RPI/EMB3119) involved in the oxidation part of OPPP lead to seed abortion beyond the globular stage, possibly due to the inability to synthesize Ru5P, and as a consequence, to the lack of substrates for purine nucleotide synthesis (Andriotis and Smith, 2019). The knock-out *rpe* mutant can develop beyond the globular stage. It is arrested at the cotyledon stage or is seedling-lethal (Favery et al., 1998; Andriotis and Smith, 2019). In our study, we obtained three knock-down *rpe* mutants. Although the accumulation of RPE is reduced to 20-50% in the *rpe* mutants (**Figures 1D**, **2B**), their growth was similar to that of wild type (**Figure 1B**, **Supplementary Figure 2**), indicating that as low as 20% of RPE can maintain normal plant growth under growth chamber conditions (**Figure 1B**). More importantly, even under high light (756 μmol photons m^-2^ s^-1^), about 75% of WT CO_2_ assimilation rate was observed in the *rpe-1* mutant with only about 20% of WT level of RPE (**Figure 4B**). The Calvin Benson cycle is co-limited by the maximum carboxylation efficiency of Rubisco (*V*_cmax_) and the regeneration of the substrate *RuBP* driven by photosynthetic electron transport (*J*_max_). The *V*_cmax_ of the *rpe-3* mutant with approximately 50% reduction in RPE (**Figure 1D** **)** was not different from those of WT, and *J*_max_ showed a slight decrease (**Figures 5B, C** **)**. The *V*_cmax_ and *J*_max_ in *rpe-1* and *rpe-2* mutants with approximately 12.5-25% reduction in RPE were reduced relative to the wild-type (**Figure 5B, C** **)**. These data demonstrate that a 50% reduction in RPE protein can reduce the synthesis of Ru5P, and to a greater extent affect the activity of Rubisco, ultimately limiting photosynthetic carbon assimilation. This is similar to the reduced sedoheptulose-1,7-bisphosphatase levels in transgenic tobacco, reductions in SBPase activity to 38% and 57% of wild-type plants decreased *J*_max_, but not *V*_cmax_ photosynthesis, and in plants with more severe reductions in SBPase activity, both *J*_max_ and *V*_cmax_ were reduced (Harrison et al., 1997; Harrison et al., 2001). These findings are also consistent with previous studies and show that some enzymes of the Calvin cycle are present at levels well above those needed to maintain a sustained rate of CO_2_ fixation (Woodrow and Mott, 1993; Miyagawa et al., 2001).

The decrease of Rubisco activity was associated with a decrease of photosynthesis only under high light conditions. Plants grown under moderate light and temperature were barely affected in photosynthesis even when Rubisco activity was reduced by more than 50% (Quick et al., 1991; De Porcellinis et al., 2018). In transgenic tobacco transformed with “antisense” *RbcS*, photosynthesis was inhibited by only 6% when Rubisco was decreased to about 60% of wild-type levels. The reduced amount of Rubisco was compensated by incubating these plants at high pH, Mg^2+^ and CO_2_ to increase Rubisco activation (rising from 60 to 100%), with minor effects by an increase of its substrates and a decrease of its product (Quick et al., 1991). The *RbcS*-antisense tobacco with a severe decline in Rubisco content showed slight photoinhibition (Quick et al., 1991). Similar phenotypes were also observed in Arabidopsis *rbcs* mutants (Izumi et al., 2012).

Why are plants with a drastic reduction in RPE and RbcS still able to maintain a high activity of CO_2_ fixation? Despite decades of research, the function and interaction of different small subunits in Rubisco is still enigmatic. Khumsupan et al. (2020) generated a set of single gene and multi gene knockout mutants for the four rbcS members in Arabidopsis, which provides a powerful tool for expanding our understanding of Rubisco structure function relationship in leaves (Khumsupan et al., 2020; Cavanagh, 2020). E.coli expression system successfully expressing active plant Rubisco and was used to analyze the plant large and small subunits of Rubisco that affect its kinetic properties, overcame a major obstacle in functional studies of plant Rubisco (Aigner et al., 2017; Lin et al., 2020). Martin-Avila et al. (2020) recently provided an effective bioengineering chassis by modifying plant photosynthesis and growth through homogenous plant Rubisco by rbcL-rbcS operon chloroplast transformation in an RNAi-RbcS tobacco (Martin-Avila et al., 2020). The excess enzymes of the Calvin cycle may be in response to metabolic and environmental changes. Post-translational modifications (PTMs) of proteins enable rapid function regulation of protein in response to the metabolic and environmental changes (Lehtimäki et al., 2015; Grabsztunowicz et al., 2017). Rubisco subunits have multiple conserved PTMs in higher plants, the conserved N-terminal acetylation of RbcL and other PTMs may be absent in the Arabidopsis Rubisco expressed in E. coli (Aigner et al., 2017; Lin et al., 2020). Thioredoxin-mediated redox regulation of enzymes in Calvin cycle has been shown to determine the carbon assimilation efficiency (Lehtimäki et al., 2015). Some proteomics-based approaches suggest that all enzymes of CBC may be subject to redox regulation (Zaffagnini et al., 2012; Morisse et al., 2014). Other types of PTMs, Lys methylation, N-terminal and Lys acetylation, Tyr nitration and S-nitrosylation, sumoylation, glutathionylation and glycosylation of chloroplast proteins have also been described (Lehtimäki et al., 2015). CBC enzymes, including RPE, are subjected to carbonylation, nitration and particularly nitrosylation in leaves, confirming a link between these modifications and photosynthetic mechanisms (Grün et al., 2006; Jasid et al., 2006; Tanou et al., 2012).

Analysis of photosynthetic parameters showed that under a light irradiance of less than about 100 μmol photons m^-2^ s^-1^, the steady state rate of electron transport through PSII (ETR II) and PSI (ETR I) are almost similar in the *rpe* mutants and WT, except for a slight change in *rpe-1* (**Figures 6A, D** **)**. These results are consistent with the normal plant growth and normal accumulation of thylakoid protein complexes under our growth chamber conditions (**Figure 1B**). However, under irradiance of 197 and 756 μmol photons m^-2^ s^-1^, the CO_2_ assimilation rate was reduced in the *rpe* mutants (**Figure 4B**). The decrease in CO_2_ assimilation may be caused by the decrease in RuBP regeneration capacity and Rubisco activity caused by the decrease in RPE level (**Figure 5**). This suggests that an insufficient amount of chloroplast RPE protein is available in the *rpe* mutants under higher light conditions. Also, the cis-element analysis shows that *cRPE* (*RPE* gene localized in chloroplasts) genes are light-responsive (**Supplementary Figure 4**) suggesting that expression of these genes is regulated by light. Further analyses showed that, under an irradiance of more than 100 μmol photons m^-2^ s^-1^, the electron transport rates through PSII (ETR II) and PSI (ETR I) are reduced in the *rpe* mutants (**Figures 6A, D** **)**. A higher value of 1-qL, which indicates a more reduced plastoquinone pool, was found in the *rpe* mutants compared with WT (**Figure 6C**). Chloroplast ATP synthase activity was also decreased in *rpe-1* in comparison to WT (**Figure 6F**), resulting in an increased accumulation of protons in the thylakoid lumen, and as a consequence, inducing a higher NPQ (**Figure 6B**). These photosynthetic properties of the *rpe* mutants can be well explained by the mechanism termed photosynthetic control (Foyer et al., 1990). During photosynthesis, reduced CO_2_ assimilation in the chloroplast stroma of the *rpe* mutants leads to an over-accumulation of NAD(P)H and ATP, which restricts photosynthetic electron flow and ATP synthase activity by feedback control. Furthermore, increased acidification of the thylakoid lumen in the *rpe* mutants limits photosynthetic electron transport at the position of Cyt *b_6_f* complex, resulting in reduced photosynthetic electron transport and an excessive reduction state of the plastoquinone pool (**Figures 6C, E**; Zhang et al., 2016).

To enhance CO_2_ fixation, some of the CBC enzymes have been overexpressed in various oxygenic phototrophs. Our study reveals that chloroplast RPE is important for photosynthesis. Thus RPE may be a potential target for genetic engineering to enhance photosynthesis. It will be interesting to test whether overexpression of RPE can enhance carbon fixation, increase biomass, and improve environmental adaptability under stressful conditions.

## Data availability statement

The original contributions presented in the study are included in the article/**Supplementary Material**. Further inquiries can be directed to the corresponding authors.

## Author contributions

YL and LZ conceived the study and designed the experiments. YL performed the experiments. YL and LZ produced the figures. All authors analyzed the data. YL, XW, LP and LZ wrote the manuscript. XW and LZ supervised the whole study. All authors contributed to the article and approved the submitted version.

## Funding

This work was supported by the National Natural Science Foundation of China (31871235), the Natural Science Foundation of Shanghai (22ZR1446000) and the Fund of Shanghai Engineering Research Center of Plant Germplasm Resources (Grant No. 17DZ2252700).

## Acknowledgments

We thank the NASC for providing the mutant seeds.

## Conflict of interest

The authors declare that the research was conducted in the absence of any commercial or financial relationships that could be construed as a potential conflict of interest.

## Publisher’s note

All claims expressed in this article are solely those of the authors and do not necessarily represent those of their affiliated organizations, or those of the publisher, the editors and the reviewers. Any product that may be evaluated in this article, or claim that may be made by its manufacturer, is not guaranteed or endorsed by the publisher.
