# Supplementary material for: Reduction in chloroplastic ribulose-5-phosphate-3-epimerase decreases photosynthetic capacity in Arabidopsis
Source: Front Plant Sci. 2022 Oct 14;13:813241. doi: 10.3389/fpls.2022.813241 (PMC9614318; doi:10.3389/fpls.2022.813241)
Supplement: Supplementary file 1 [file DataSheet_1.pdf]

FIGURE 1

1A

Sequencing results of T-DNA insertion sites

rpe-1 (T-DNA LB-Sequence/*RPE*-Sequence)

TTTGCTTTCCCCCTATAATACGACGGATCGTAATTTGTCGTTTTATCAAAATGTACTTTCATTTTATAATAACG
CTGCGGACATCTACATTTTTGAATTGAAAAAAAATTGGTAATTACTCTTTCTTTTCCTCCATATTGACCATCAT
ACTCATTGGATTAACCATTTACAAGGAAGACGTTACCCACGAGACCGTAATCACACGTGGCGGATTCTCAG
ATATTCTCTCTTTTTCTTCTTTACCCGGAAAAATAAAGTTTCTGTTCTTCTTTTTTCTTCTCTGGGTTTGAGAA
TTGGTGTAAGAGAAACGATTGAGGTTTTCTCTTATTATCTAAACCCACCAAGTCTTTCAGGATTTTAGCCAG
AGAAGCTTGAGTCTTTGATTAGGGACATGTCAACCTCCGCCGCTTCCTTGTGTTGTTCATCAACCCAGGTC
AATGGGTTTGGTCTTAGGCCTGAAAGGTCGCTTCTTTACCAACCCACTTCCTTTCATCTCCAGGTAGCTTTA

rpe-2 (T-DNA LB-Sequence/*RPE*-Sequence)

TTTTGCTTTCCTTAATATATCTTCCCAAATTACCAATACATTACACTAGCATCTGAATTTCATAACCAATCTCG
ATACACCAAATCGAATTCAATTCGGCGTTAATTCAGTACATTAAAAACGTCCGCAATGTGTTATTAAGTTGT
CTAAGCGTCAATTTGTTTACACCACAATATATCCTGACGAGACCGTAATCACACGTGGCGGATTCTCAGATA
TTCTCTCTTTTTCTTCTTTACCCGGAAAAATAAAGTTTCTGTTCTTCTTTTTTCTTCTCTGGGTTTGAGAATT
GGTGTAAGAGAAACGATTGAGGTTTTCTCTTATTATCTAAACCCACCAAGTCTTTCAGGATTTTAGCCAGAG
AAGCTTGAGTCTTTGATTAGGGACATGTCAACCTCCGCCGCTTCCTTGTGTTGTTCATCAACCCAGGTCAAT
GGGTTTGGTCTTAGGCCTGAAAGGTCGCTTCTTTACCAACCCACTTCCTCTCATCTCCAGGTAGCTCA

rpe-3 (T-DNA LB-Sequence/*RPE*-Sequence)

GGGCATCGCCCTGATAGACGGTTTTTCGCCCTTTGACGTTGGAGTCCACGTTCTTTAATAGTGGACTCTTGT
TCCAAACTGGAACAACACTCAACCCTATCTCGGGCTATTCTTTTGATTTATAAGGGATTTTGCCGATTTCGG
AACCACCATCAAACAGGATTTTCGCCTGCTGGGGCAAACCAGCGTGGACCGCTTGCTGCAACTCTCTCAG
GGCCAGGCGGTGAAGGGCAATCAGCTGTTGCCCGTCTCACTGGTGAAAAGAAAAACCACCCCAGTACATT
AAAAACGTCCGCAATGTGTTATTAAGTTGTCTAAGCGTCAATTTGTTTACACCACAAGATAAGCCGCTGCAT
TGGATCATCCGAACAAGGAAGACGTTACCCACGAGACCGTAATCACACGTGGCGGATTCTCAGATATTCTC
TCTTTTTCTTCTTTACCCGGAAAAATAAAGTTTCTGTTCTTCTTTTTTCTTCTCTGGGTTTGAGAATTGGTGT
AAGAGAAACGATTGAGGTTTTCTCTTATTATCTAAACCCACCAAGTCTTTCAGGATTTTAGCCAGAGAAGC
TTGAGTCTTTGATTAGGGACATGTCAACCTCCGCCGCTTCCTTGTGTTGTTCATCAACCCAGGTCAATGGGT
TTGGTCTTAGGCCTGAAAGGTCGCTTCTTTACCAACCCACTTCCTCTCATCTCCAGGTAGCTTCA

1B

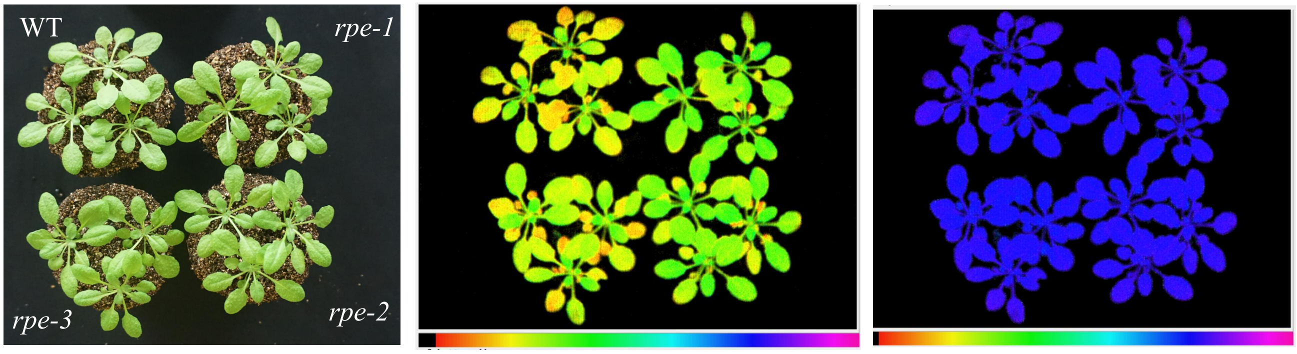

1D

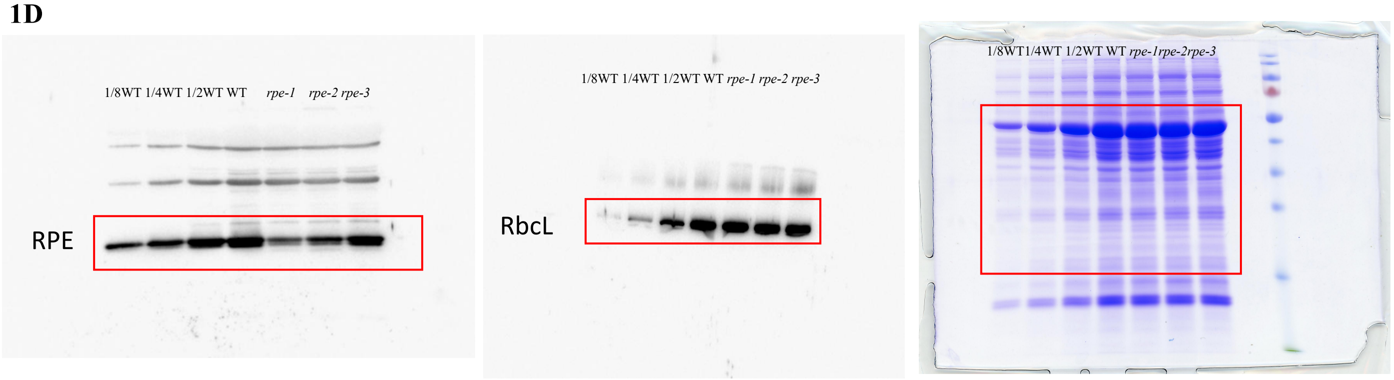

FIGURE 2

2A

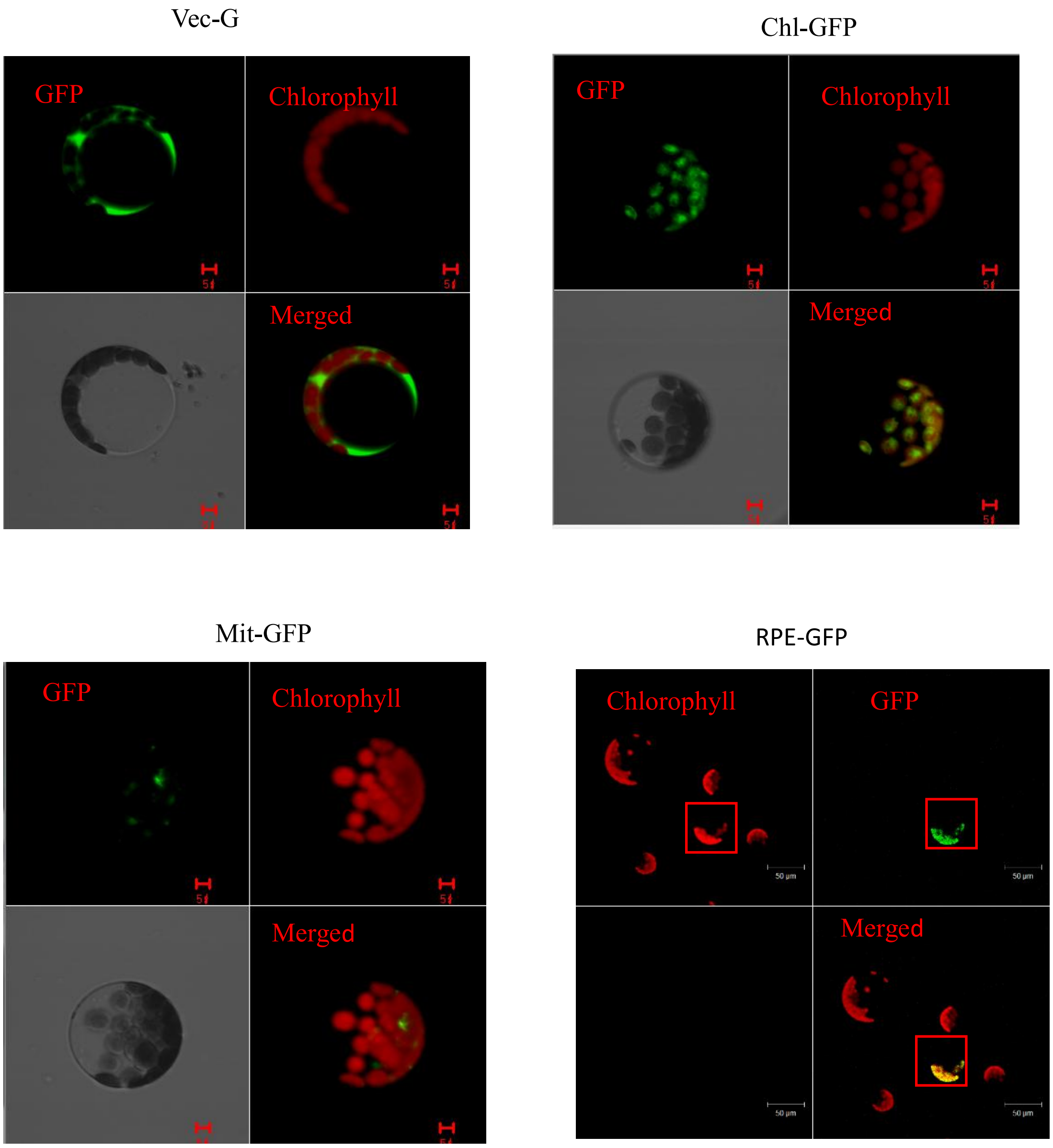

2B

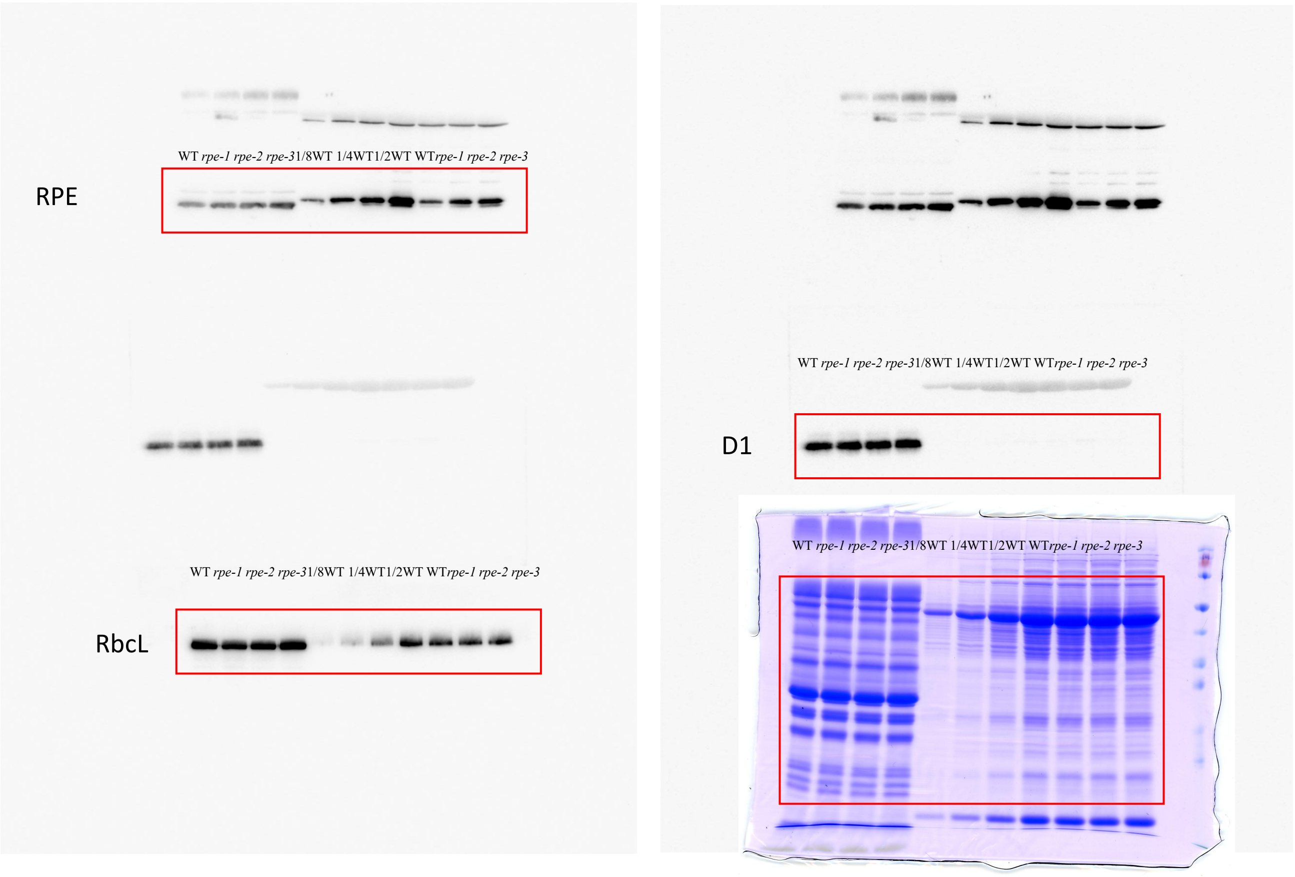

FIGURE 3

3A

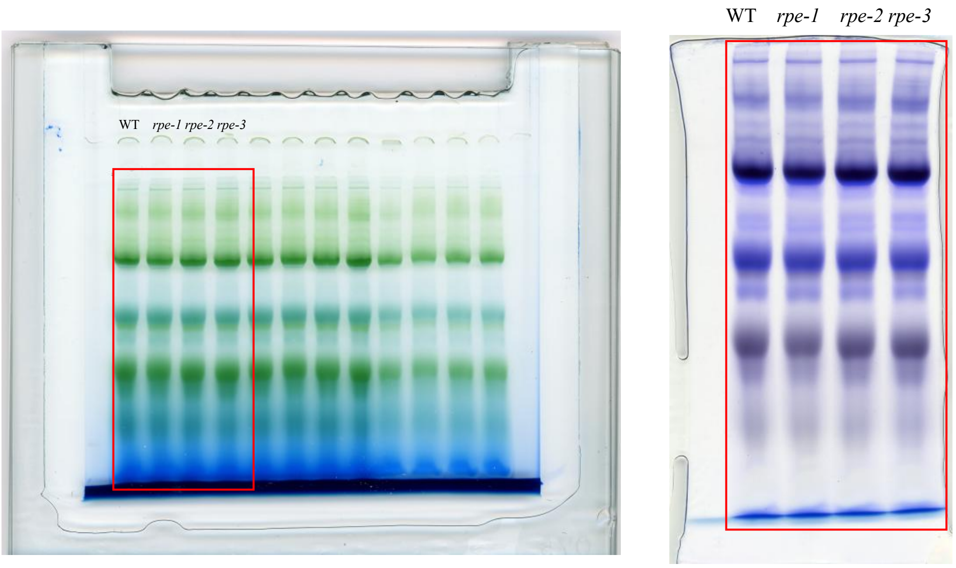

3B

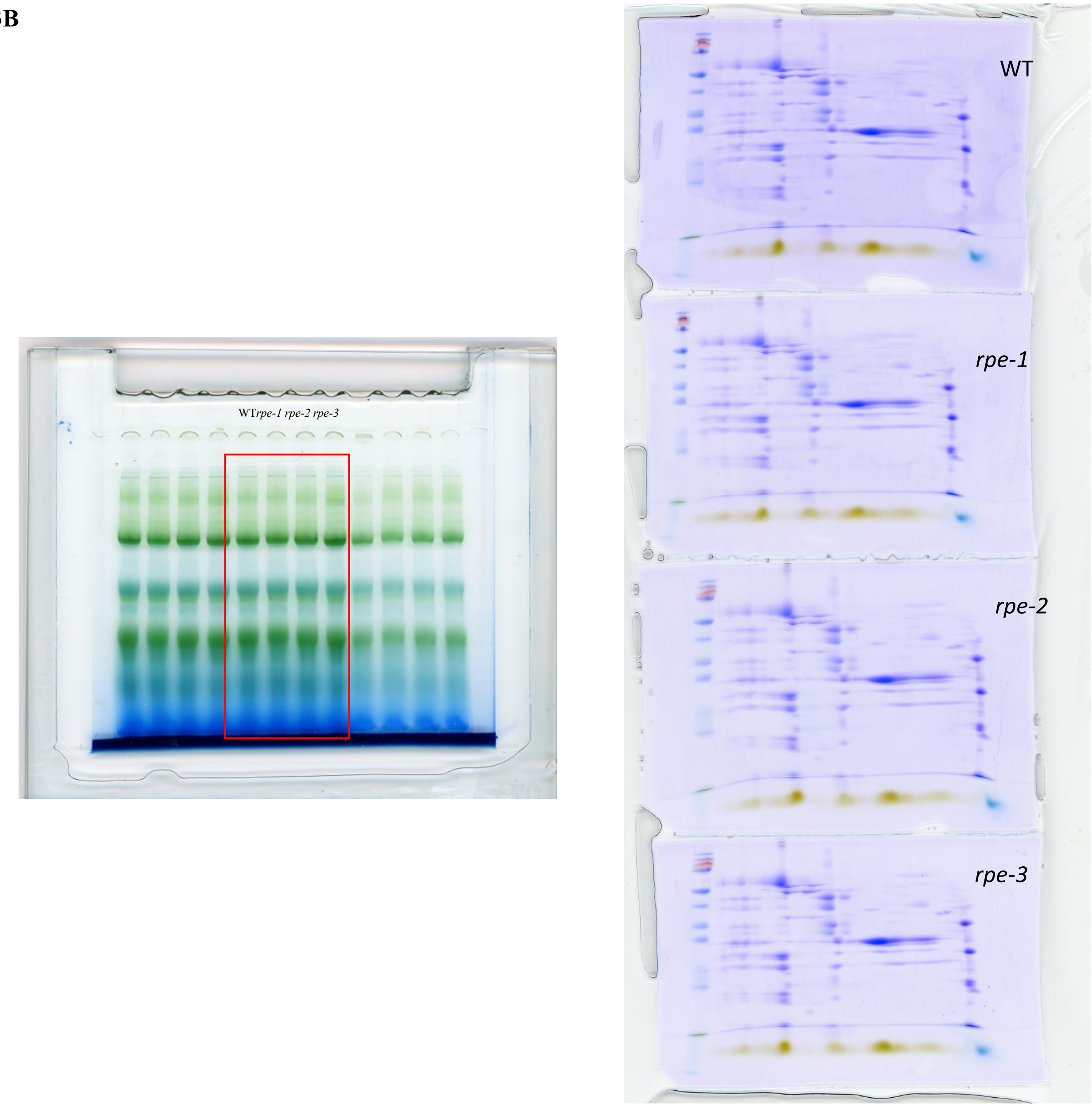

3C

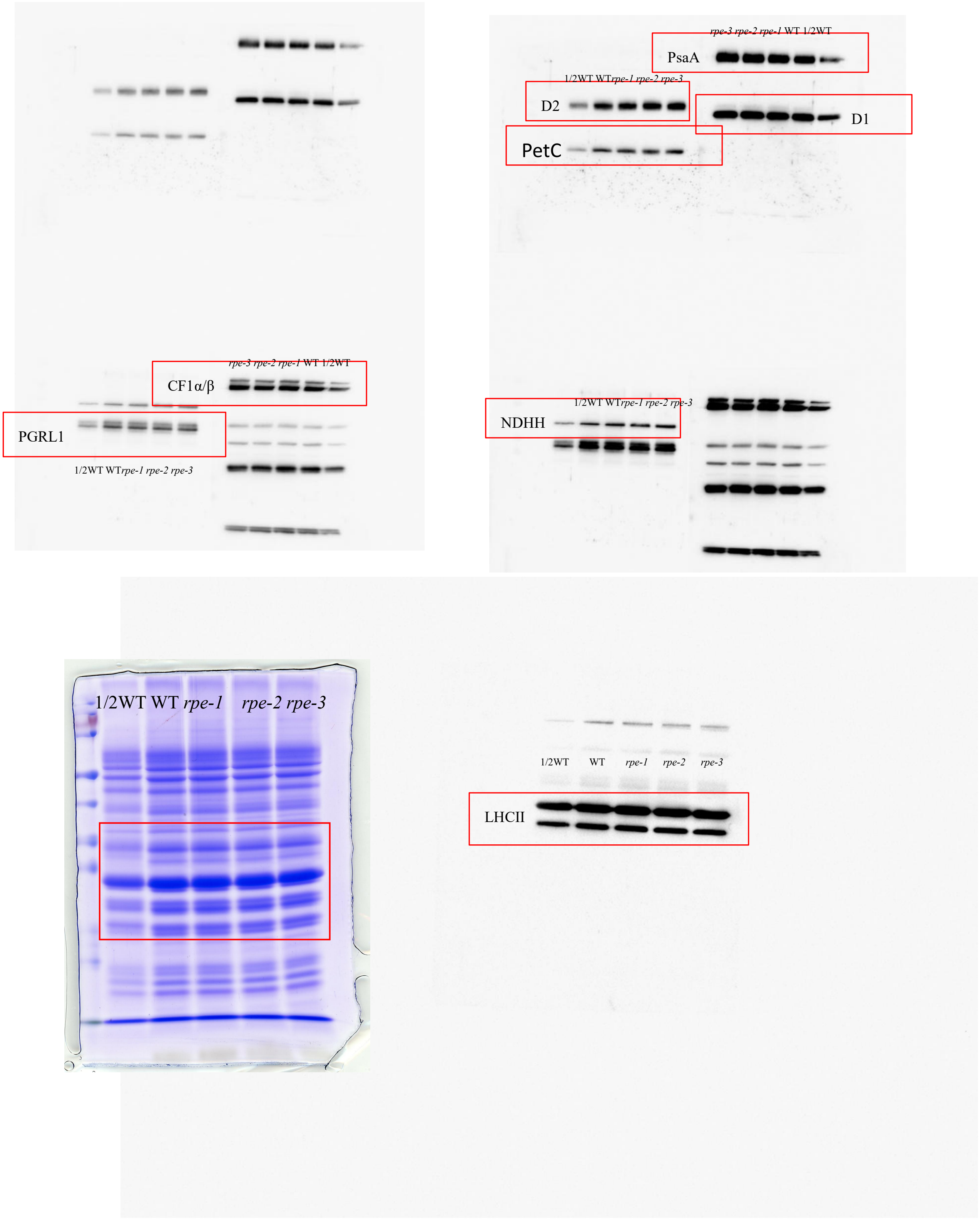

rpe-3 rpe-2 rpe-1 WT 1/2WT
CF1α/β
PGRL1
1/2WT WT rpe-1 rpe-2 rpe-3
rpe-3 rpe-2 rpe-1 WT 1/2WT
PsaA
1/2WT WT rpe-1 rpe-2 rpe-3
D2
D1
PetC
1/2WT WT rpe-1 rpe-2 rpe-3
NDHH
1/2WT WT rpe-1 rpe-2 rpe-3
1/2WT WT rpe-1 rpe-2 rpe-3
LHCII

FIGURE 4

4A

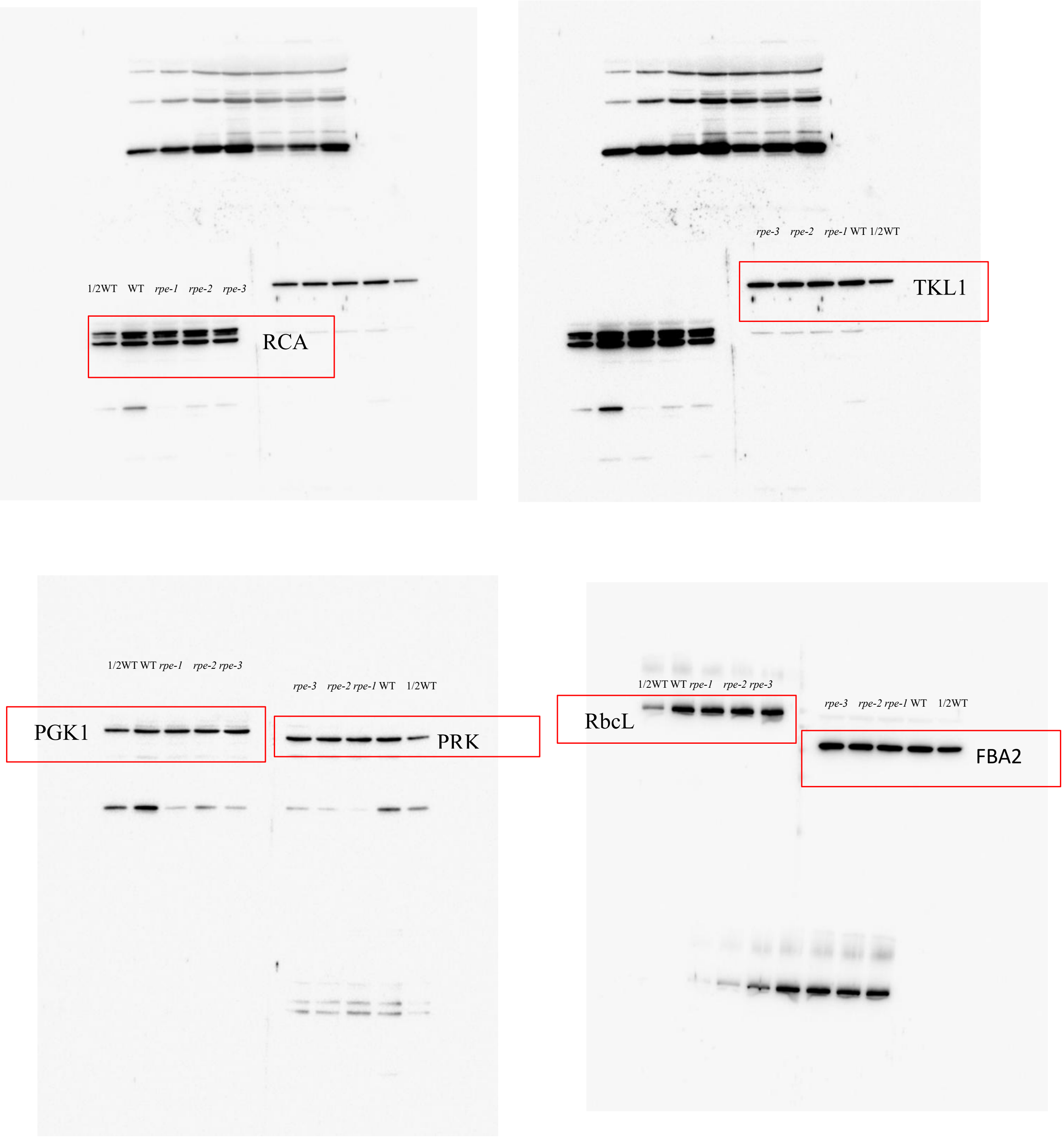
1/2WT WT rpe-1 rpe-2 rpe-3
RCA
rpe-3 rpe-2 rpe-1 WT 1/2WT
TKL1
1/2WT WT rpe-1 rpe-2 rpe-3
PGK1
rpe-3 rpe-2 rpe-1 WT 1/2WT
PRK
1/2WT WT rpe-1 rpe-2 rpe-3
RbcL
rpe-3 rpe-2 rpe-1 WT 1/2WT
FBA2

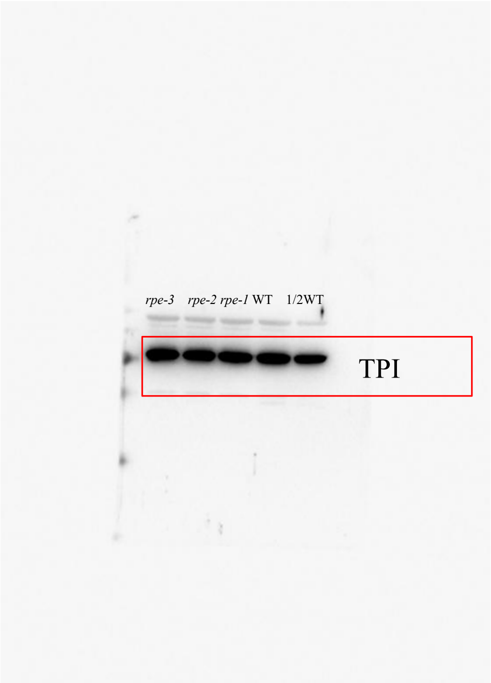
rpe-3 rpe-2 rpe-1 WT 1/2WT
TPI

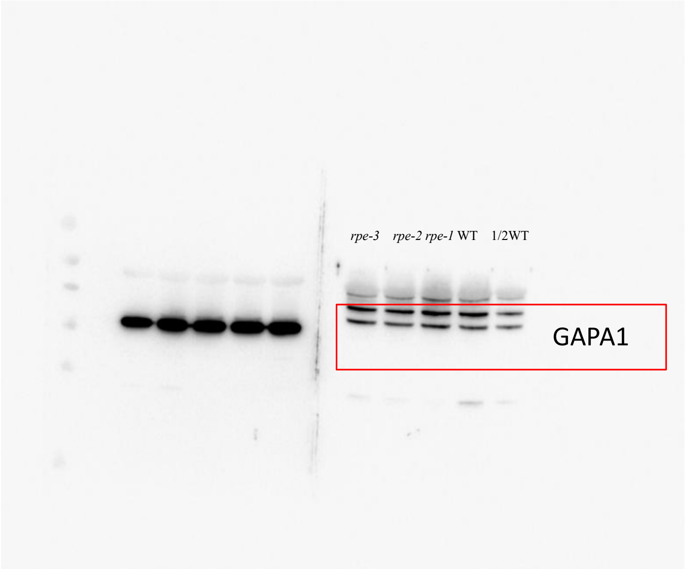
rpe-3 rpe-2 rpe-1 WT 1/2WT
GAPA1

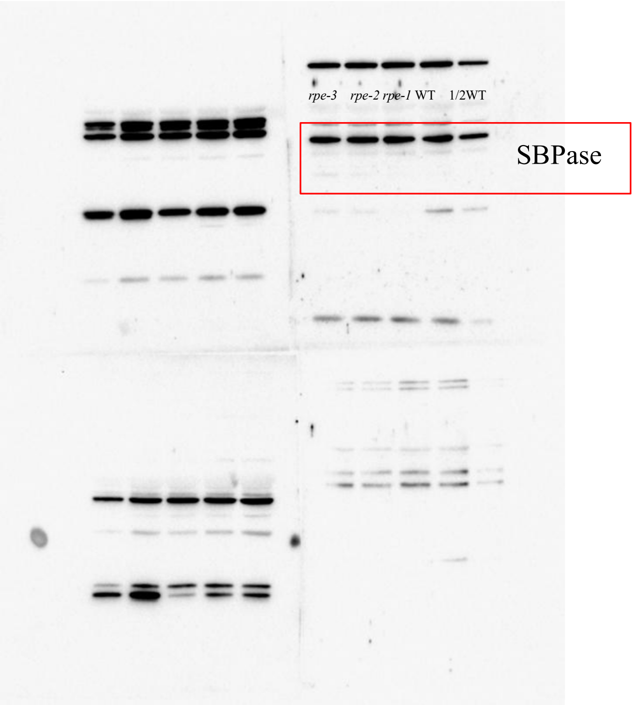
rpe-3 rpe-2 rpe-1 WT 1/2WT
SBPase

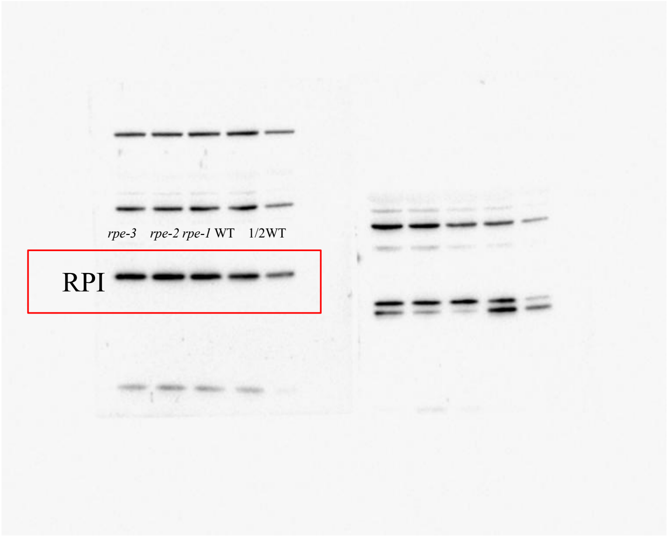
rpe-3 rpe-2 rpe-1 WT 1/2WT
RPI

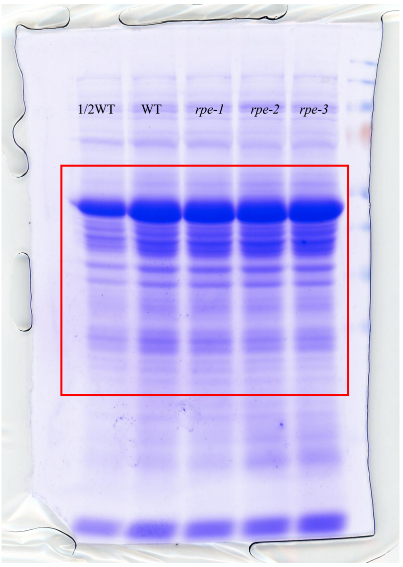

Supplementary Figure 1

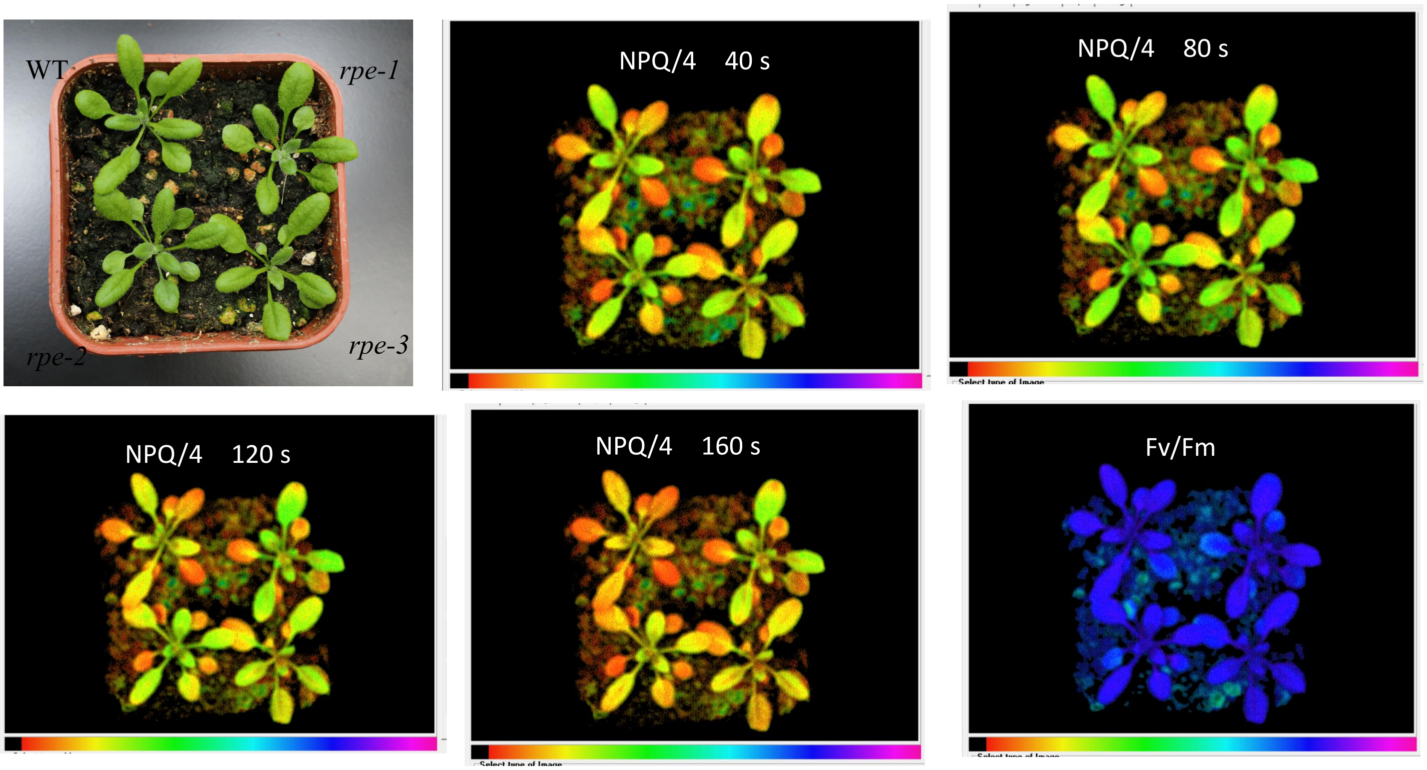

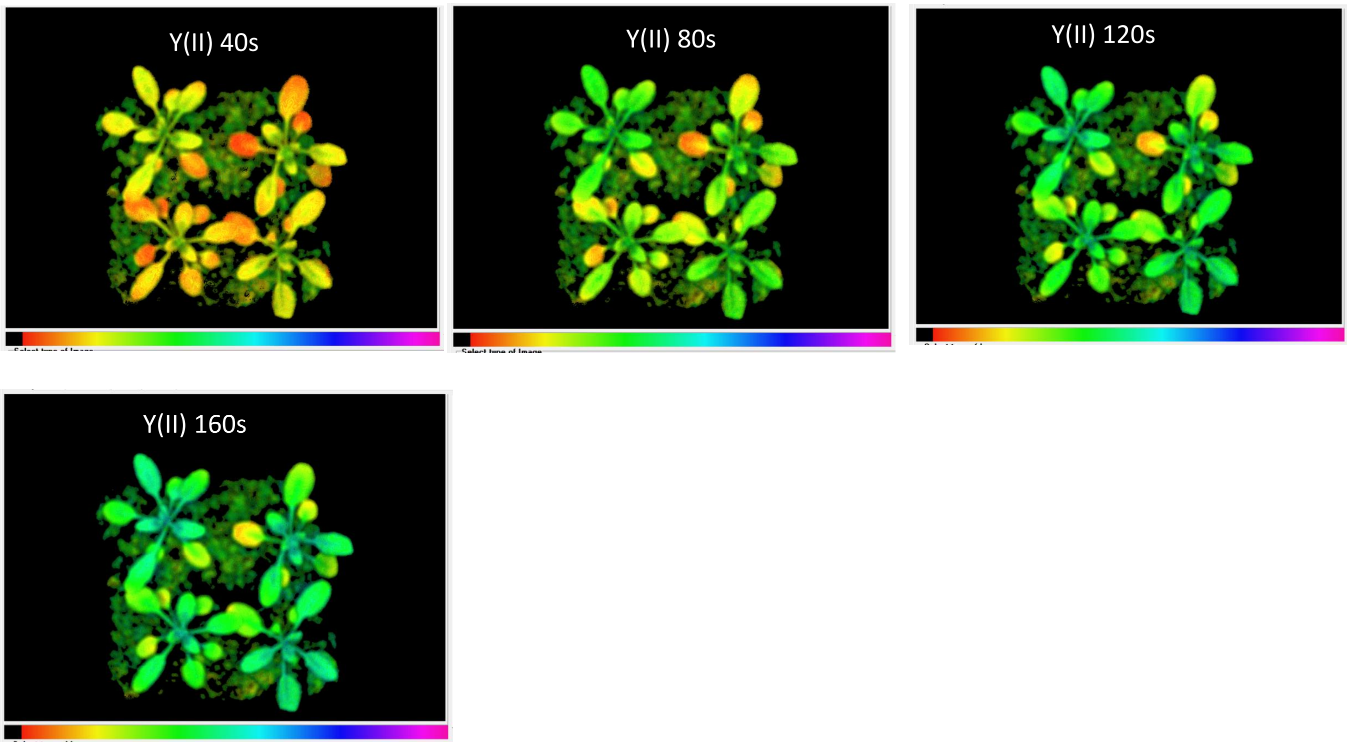

Supplementary Figure 2

2A

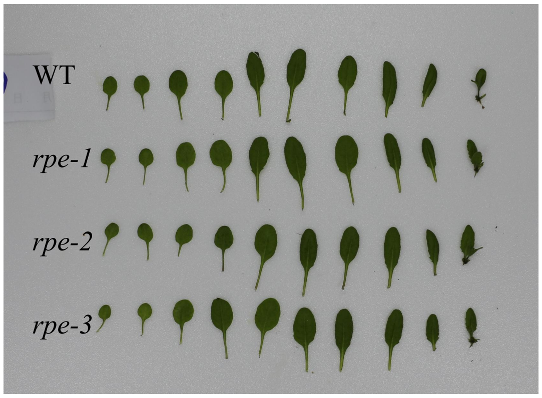

Supplementary Figure 3

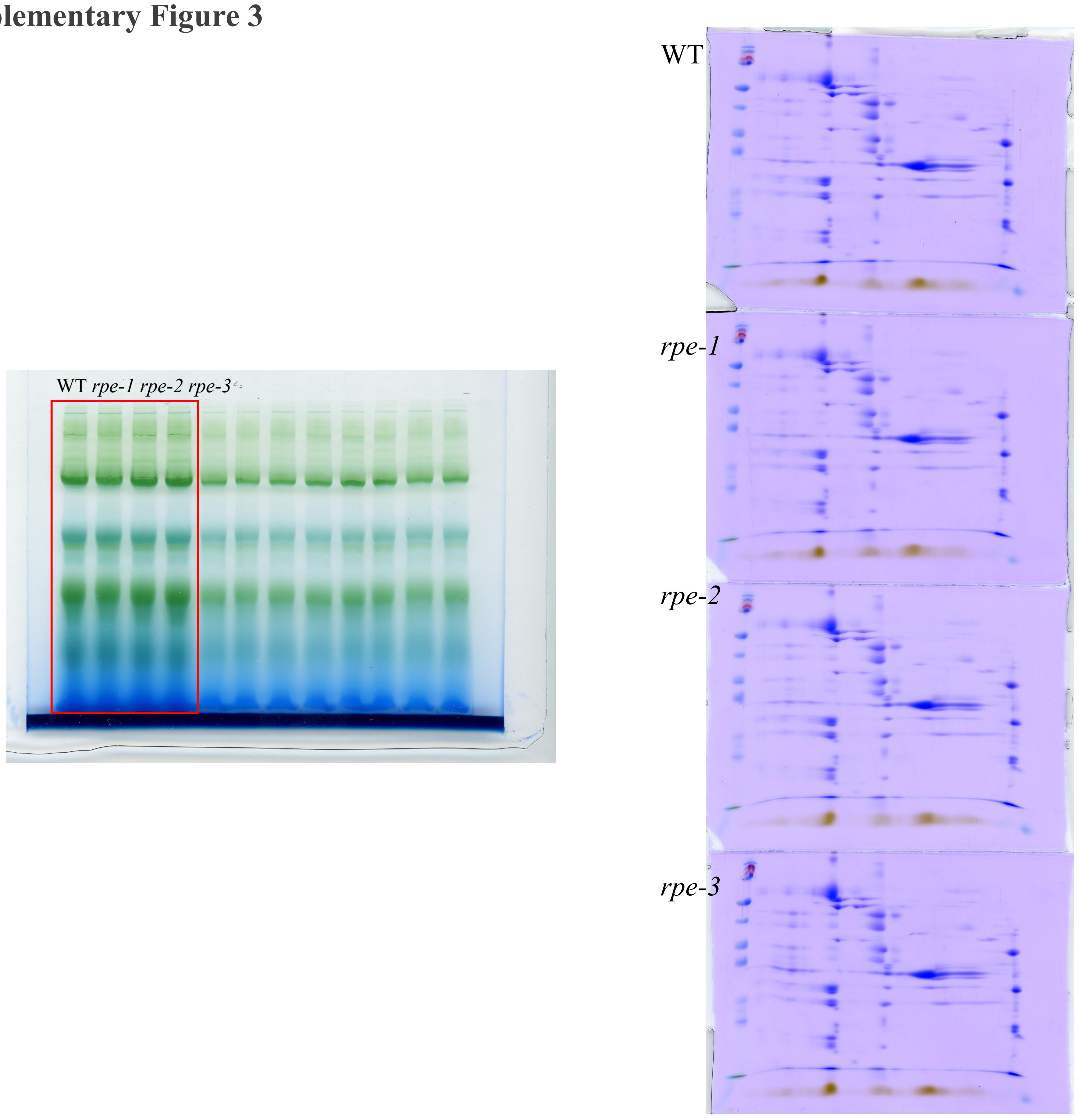

Supplementary Figure 4

Sequence of the 2 kb upstream region of cRPE genes

> Cre-cRPE Cre12.g511900.t1.2

GCATCATGGCAAGGAGTGCCGCTGTGTGTGTCGAGGTGTGCGCCGACATGATGCTCACGGGCTGTGGCTCGATAACCACAACTCCT
GTTGCCCTGGCCCCCGCAGTGGCTCCTCACCCGAGGGCGTCACGCTGGTGTCAGCCGAGAACAGCACTGCCGTGGGCGTCGGCCC
CGGCGCCACCTCCGCCACAGACGGCTTTGGCAGCCTGCCGACGCTGCTGGCCGCTGGAGACGTGCCGCACGCGATGTTCTACACCT
TTGCCGCGGGAGCTGGCAGTGCAGATGGACCCAGTACTGACAGCGGTGGCGGTGATAGCAGTGGCCGCGGCACCAGCGGCGGTA
GCAGTGCCGGCCAGGGCGGCCCCGGAGAGAGCACTGGAGGGCAGCAGAGCGGCCGTGGGCGCACTAGCCGTGGAGGCGGTGGT
ACCGGCAGTAGCAGCCGTGGTGGCTCTTCCAGCGGCGGCAATGACCTGAATACGGTGCAGCTGCCAAACTGCAGCTAAGCGGGCG
GCTGGCGCTGACCCCTGGCGCTTGTAAGTGAGCTCGGTATGCGCACGGGCGAGCTCAGGCTGTGTGTGTAGGGTGACATGCATTTC
GTGCATTGTGCATGCGCGCAGCCTGCATGCACTAACACATTGGCGGTAGGGGTGCGGCGATGTGCACAGGACACCGGACACGTCGG

GTTGGTGGAGTTGCTACGTTGCAGATGACAGGTTGCAAGAGACCGCACAATGCGTCCAGGGGATTACTCGTCGGTAGGCCGTGCGT
GTGCGCGACAGTGTCTGCGGTGGCAACCCTGAGGGGCTACGCGCCAGTTGCGACAGGCTGCGAGCCCATTACGGCGCCAGGATGT
GGCCGCAAAAGAGCGCGGCGGCTGGACGGGCCAGCGGCTGGAGATCGGAACTTGTTCGAGTGTTGACTGTTAACGGTAGCCCAA
AACAAATTGAGTCCAGCACGGGCCCGCCACGGCGAAAGTTACTACAAGCCCTTGTCAGCTCCACTGCTTCACCAAGTGTGGGTATGC
GTGCCACAGTGCACACACAGGCTCGACAGCAGCTGAAGTGCTGAAATGCTAAACAGCCTGCCTGAAATCACTTGGCTTGCCCGGAC
TGCTCCAGTCCAGTTGCCCAGTCACAGCTCTGGGTTGGACACAGAAGCGGTATGCGAGTGACCTGGCAACCTGAAGTTCACATCCA
CCAGCATCAAAGCTGTATGCGGGCAAAATGGTATGGTCAACGTGCTGCATGAACTGGGACCAAATGAGTTGACCCTCGCCTCTACCA
GCCACCACTCATCATCAGTTGAGAGTGGAGACCAACTCGGGACCCACATCGAGCACCCCCCCCAGGCCCCCACCCCCCCGCACACA
CAATGCCCTTACAAACTGCATTGCCGTTCCATCACCAATATCCTGCCCTCCATGTGCCAATTCACTTGGCTAGTTGAAATGCATGCGCAC
GACACGGAAGCGCCCGCATACGGGTTGCGTTGGGCTCGGCCCCGGCGCAGCAACCCTTGCAAGGCACAGTGCATGGCTAGGTGCC
GCGGCCTCCTCGCACAGCCCTTTGCCAGGGGTACCCTTTGCTGTCGCCCTTGTCAACCACGGGGGCACCATTTCCGTGTCAAAGTGC
CACCGGCACATGCGGGCTGCTATAGACCTTTGTAGGGGCTTTCATAAGAGGAGAGGCTCTGACAGGCAGGGGGCTGGAGGGGTGT
CAAAGCGTGGGCGCGTGGGGCTTATGTTGGGGCCCCACTGCTCTTGAAGGAAACTGAAATGCAGTACTTTTCAACAGCTACTCAAAT
GGAGGCGGCATAGAACATAGAAGAGCATGCAGCAATTTGTGCGCACTGGAACCGAACGAATCCAACAAGGGATAAATACGAGCGG
GCGTTTTGTCTCAATCCCGGCGATGAGGCACATGACGGGGTTGAGTCCCGGTCGACTACCTCGCGCAGCTCTGTTACGTCGCTTGTT
CGCCGTTACCGTTCAAACCCAGAAAG

> Pp-cRPE1 Pp3c1_8270V3.1

TTTTGGCTATCATGTGAAACAATCAACCGGAGAAATTACTGCAAATATTATGACCACTTGATTTCTAGAGACGTCGAAGATGTCATTAG
GTCTTAATATCGGACGCCTATCTCTTGCGTAGTTCTTGGTAATGAGAGGCTATGATTCGTGATTGCACAGATAGCTTTGATTCCTCAACG
TTTGGAATGCTATGGTTTAAAACTTGCCGATCGCTTTGCCACAACAATTCAGGTCGCACAATGAAGCACACCATGATATGCAACAGTA
ATGGTTTCTAACGACTCGTTTTACATCTCGACAGGGAAATGTCGGTCTCATCTTCACGAAGGGAGACTTGAAGGAAGTCCGTGAGGA
GATTGCCAAGTACAAGGTCGGAGCACCTGCTCGTGTGGGTTTGGTTGCCCCCATCGATGTGGTTGTTCCCCCCGGTAACACTGGTCT
GGATCCCTCGCAGACATCTTTCTTCCAGGTAAGGTCTTCGGTATATGGTATGTTCTATGAATGAGATTCCAGTCAGTCAATTTAGCTTAT
CGCAGTGGGCAAGTGTCCTTTTTCTGATGTCGTTCTTTATGGCAGTTATGAAACGCTGTTAACTGAAGGTTTCTGACGAATAGAATGC
TGAACTCTCCAGGTCCTTAACATTCCCACCAAGATTAACAAGGGTACTGTCGAGATTATCGCCCCTGTCGAGCTTGTCCACAAGGGTG
ACAAGGTCGGATCGTCCGAGGCTGCGCTTTTGGCGAAGTTGGGCATCAGGCCATTCTCTTACGGTCTTGTCGTTGTGAACATCTACG
ACAACGGTTCAGTCTTCGCACCTGAGGTGTTGGACCTGACCGAAGACGACCTGTTGGACAAGTTCGCTGCTGGTGTCTCCACTGTG
GCTGCAGTGTCACTGGCCCTGAACTACCCCACCTTGGCTGCTGTCCCCCACTCCTTCGTCAACTCGTACAAGAACTTGTTGGCTATTG
CTGTGGAGACTGAGTACTCTTTCCCTCTTGCTGAGAAGACCAAGGAGTACCTCAAGGACCCCTCTAAGTTTGCTGTAGCAGCTGCAC
CTGCCGCTACTGAGTCTTCATCTGCTCCCGCAGCTGCTGCCAAGGAGGAGGAGAAGGTTGAGGAAAAGGAAGAGTCTGACGATGA
CATGGGATTCAGTCTTTTTGACTAGGACGGAGTAGGTGCATGGTGTTGATATCGGGGATGTTATCGTCCACTAGTGGAATGCTCTGAT
TCATTTGAGAATGTACTGCACGCTTTTTACTTACAATGGCATCCACTTGATTACGCTTTGCAGCGCTTTTCAGTATTTACTACATTCAGT
CTTGTGTTGGACGTGTTGGTGACATGAGCCGAGGCAGTCGCTTCCACTTCCGTTGAATCTGTTACTGACAAGGGAGTACAATCCCTCT
ACATGGTATTATTGTATCGTTGTGAATCTTCAGAGCCCCATTTAAGCCTTATGTAAGGACGGAACAGACTCTCTGACTTCGCATTTCGTC
ATGTACAGTTTCCTAATTCATTTTCCTTCTATCAATCTTGGTTGTGTATGATCGTTGACGTAGACAGTTTCATAGCATTAAAGCAAGAAA
AATAACAATAATTATAATTCTCACCTTCCTAGCACACACAACTTTGAAAATATATAGATTTTGTAACAAATATAAAAGGTATATATCTATATA
CTCGGAAGAATATTTCGTTTTTGTTAGTTTACTCCTATTTAGTTCAGTGCACTTCTCCTCCGTTTCACGTAATTTTATTATAATTAGTCACT
TCCTCGAACTTCACTGAAATTTGTGTATGTATATCTACATGTACAATTTGTATACTACTGGAATTTTCGACTGTAAGAAATGTGTCCACTG
ACAGATATTACTCCTTCAATAGAGAACGCCACTAAATAGAACAACTATGTAAATTCTTACTCAGACCGACGAAGATAGAACAAGATCCG
TCACCACTAGAGCACGTAGCTCCCATTAACCCGACGAATCGCCAGGGAT

>Pp-cRPE2 Pp3c7_23810V3.1

TATATATATATATATATATATATATAGTTATTGAGATCCTTAATTTAAACAATATAATCCTTATAGCTTAACTTGATCTTTTTAGTCTTCTTTTTGTGC
CTTGTTTTAGCTCTTTGTTTGTGATATAAAGTAAATTTCTTGTGTTTTATTTCCTTAAACTCTTGAAATCTTATCCTACTTGAATGGTATAT
CCATAACATTAAGTCCATTAGAATTGCTATTATTTAACTTAGAGCTAAAGGTTGGATATTCAATTATGTTGTTGCATAATATTTCAAACTTT
GCATCTAAGGCTTTACAATGGATCATACATGGTCATTATAGATCTCACTAATTGAGTTATAGAGACAAGACTATTAGGTAGAGATTATAA
GGAATAATTAATTTTTCTTCTACCAATCTTATTATGCCCATCATATACACAAGTTTTGTTTTAGTTCAAGCAAAGGTAATTTTCAATAAAG
ATAGCATTTATAATAGCAATAAACAAATTTTAAAGACAATCATTGAAGAATATAAAAATAAATCTTAAAATCTCTATATTTATACAACAAA
AATTATATGTTACATTTTAAAATTATATATTGCATTTTTGTGATACAATTCTCTAAATACAATAAAGATCCTTTATTATCAAGAAAGTAAAG
ACAAGATACCAATGTAAATATAATTTATTCTAATTTTTTTTACCTTAATTATTTTAGTTGTTGTCATATGTATATATATATATATATATATATAT
ATATTTATCAAGACATTGAAGAAATATAAATGATGCTCTCGTTATGAGTATTTTATTTTATTTTATTTTCATTTTCTTAGGTTTGATGTTTCT
TTAGGAGTTATCAATTTGTAACTTAGTAACCTCAAATTAAAAAATTTACAACCTATTTGTATCCAACAACATTAGTTTTTGTTAGTGCCTT
GGTGAAGTTTCTATCCATTAGTCATGTTCTAGAGGTGATTTAACCAAAGCAACAAGAAAAGGCCTCCATGTTATATATAGTTGGTCTCT
AGTTAGTTGGCCTATATTATGTTTAGTATGATTGTGGTATTTCATATTTATTTTTTATTTAATTAATAATATAATTTAATAGAAAAATCATATA
TCCCTTATGTTTCAAATGAACAAATATACATTTCACTTTTAAATGATGGTTGTCTTTACTTAAGCAATGTGAAAATATATTACACATGTGTA
TTATTAACACATGACTTAGTAACCACAAATTAAAATAGTTACATCCTTTCAATGTCCAACCAACTGCACTGCTTTGTACTGTAATAAATAA
ATAAATTTATTTATTTATTACCGTTGGAATATTACAGCACCGCATATTGTGACTGAATGAAGGAATGAAGGTGCGCGGCAATATTAGTG
GTCGGCGGTAATCCTCGAAGAAGATTCATGTGAAAGCGGTGGCAACTTCGATAAAAAGTTCAATGCTGATTCACTGGGTAATGAATG
AATGATTGAATGAGCAGGTTGCATCGTCAAATGACCAGCTCCCAGAGATATAGCTCGGGACAAAGATCAGAATGCGGAAAATGCTGA
AGAATATGCTGAGTTTTAGGGCGTGAAAGATGAACGAGAATTCAGAATGATTGGTACTGGCATATAACGTTTACTTGGACTGACAGC
GGCAGAAAGAAACCCAGATGAAACGGGGAAGAAATCGTTGTAAAAGTTTGATCGGCGTTGGTGGGTGTGAGGATGGCAGATCCTG
GGCCACCTGTTTTCCAGATGGCTTCGTGAGACTGTTTGTGTGCGTGCCCTGCCGTGCGCTTTTTCTCCCTTATCCTGTTCAGCCACGCT
CAACCACGCTTCATATTTCGTTACAAGACCCACAAGCCACACGCTGATTAATGTATATAAATGCGTTCGTGTCCTGACCCATGTTCTAGT
TACATTTTAATTCTCTCAATTCCGCCCCAGTATTTAGTGTGCGAGCAAATTTGAATTGCGGGGGTCCTGAAGTCTGTTGTAAAGTTGCA
AGGAGCG

>Pp-cRPE3 Pp3c11_3710V3.1

GGTGTTTCGAAGCTAAAAAACACATTCTGGAATAGTTAACATGTTGAAGTGGAATTTGTTGAAAGCCTCTTTTCAAGCCATTAGAGGC
TTGAATTGGAACAAGTTGTTCTTACCAATGTTTGCCCTCATTTTCAACAGGGCAATGTTGGCCTTATCTTTACCAAGGGCGACTTAAA
GGAAGTCCGTGAAGAGATTGGAAAGTACAAGGTCGGAGCTCCTGCGCGTGTGGGTCTTGTTGCCCCCATTGATGTGATTGTACCTCC
TGGCAACACTGGTCTGGATCCCTCTCAGACTTCTTTCTTCCAGGTGAGATTTAAATTACGCACTTGATGGCAGATATCATGTTTTCACA
GTGACATCTGATATTTTGTCCACTTTCCGTTTCTGACACCTGATGCGTGTTGATTATCAGGTCCTCAACATCCCCACGAAGATTAACAAG
GGTACTGTCGAAATTATTGCTCCTGTAGAGCTCGTCCACAAGGGTGACAAGGTCGGATCATCCGAGGCTGCTCTTCTTGCAAAGCTT
GGCATCCGGCCATTCTCCTACGGTCTCGTTGTTGTCAACATCTACGACAATGGATCCTTGTTCGCGCCGGAGGTGCTGGACCTGACCG
AGGATGATCTTTTGGACAAGTTCGCTGCAGGTGTGTCCACCGTGGCCGCGGTGTCTCTGGCCCTTAACTACCCAACCCTGGCTGCTG
TTCCTCACTCCTTTGTCAACGCGTACAAGAACTTGTTGGCTATTGCCGTGGAGACCGAGTATTCTTTCCCCCTCGCCGAGAAGACCAA
GGAGTACCTCAAGGACCCCTCGAAGTTCGCAGCTGCCACTGCTCCTGTCGCTGCTGAATCTGCAGCACCAGCAGCTGCTGCGAAAG
AGGAAGAGAAGCCCGAAGAGAAGGAAGAGTCTGATGATGACATGGGTTTCAGTCTGTTTGATTAGAATTCCTTGGAGCTTGAGGTT
TTTCCCTGCTTTACTTGGCAGTCGGACTGATGCCCTCGAAAATGTATCGTTTTATCCTACTGCTAGTAAAGGTCATTCTCTTGGCGAAA
CGTCTTTTCCCCCTTAAAATGGAGTCTGTCCCGGTACGCTGCTTTGGCCACTTTGCAGCCAAGTGAACTTGTTCTTGTCATCTGCATTG
ATTGTCCATACAAAATAAACTACGACTCTGTCTTACTAGTATTACCAATAAGATCCAATGTAGACGACCAACTCCACGTCAATATTCTCCA
GTGCAATAATGCAGCATTCTAATCATGTTTGCGAAAATTTCTTGTCTATGAGTGCTGTGGCAGACGCACTAATCCATTATACCACAACTA
CATCGCACCATAGCTGCTGCATGCATTCAGAACGCTCCTTTTCATACCATTGTACCATTCAATATCAAGCCGGTAAAAGTATGGGTGGA
GGCAAGCATGCTTAAACGGTGTTCAGTTTGGGCTAGTAAAACCTGATGTGCCTACACATCACTGAAGATATGATATCATCGAATGATG

GGCTTGCATCGAAAATAGCTAAGAAACCACCAGTACGAGGGGAAATTCCAAGCACATTCAGCAAATTCGTTCCAGAAAGATGAACG
AGAATCCAGAACCATTAGATCCGGCGCACGATGTGGATTCAGATAACTGCCAAGAACCCAATTCAGACAAAATTGGGCCGAAATCGT
CGACCATCTGGACGGACGTTGCTCGTCCAAGGTTGGCAAATCCTGAGCCACCTGTTTCGAATTTGGCTTCTGGCGGCCCTTTGGATG
CGTTCCCAGCAATGCGCTCGTCCCTCCCTTATCTTGTCCAGCCACGCTCATCCACTCTTCCCATGTCATTCCAAGTCCAGCGAACCACA
CACAGATTTGCGAGTCTTCCTCAATTTTTCAATTACCTGGCTCAGCTCTATTCTCCCTTAGCAACCTCCGTCTCGACCTTGGTGAGTGTG
CCCAGTGTGTGCAAGGAATTGAATTGCGAAGTTGGGTAGGCTGTTGAGGAAGCAAGGAGAG

> Sm-cRPE 228435

GCAAGGAGAGGATGATGCTCAACGAGCGCGTGACGCTCCTGCTACTGAAGCTGGACGAGATCGACGCTGTCCAGCCCTTCGTCAAG
GAAGTGAGGAAGGCCTTGATCGTGGAGCTCCAGCAATTCTCGAAGCCCATGGAGGACAAAGATCGAGAAGAGCACGAGAAGAAC
AGAAGCAAAGCTCAGGACATCCAGATCGAGGACGCGGAGGATGACCGTGATGGAGAAGAGGCTCAAGACATCGAGATCGAAAAC
GCGGAGAACGATCAGGACGATGATTTCTTCATCGTCAAGCACGGCGATGCCGAGGACTCGATGGACACCGAGCCGGAGAGCACGA
TCTCGGAGGAGGAAGATCTTCACAGCCCCGAGAAGATTGATAACCAGGAAGTTCCAGTGTCGAGTGAAGCGGTCGAGCAAGATCA
AATCTCCGGGCAAACAAGCATGGACGTGGACGTGGTGAAGAAGCTCATGGAGGACAATGCGCAGCTCAAGGCGATGGTGGCCAAG
TGCCTGGAAGGGATCCAGTGGCAAAACGCCACCATCGCCAAGCTTGAGAGGAGAGTCCACGACTTGGAGAGAGCTCTTCTAGACT
CTTCTAAGACTGAGAACTCGCAATGTTGGATAAGTTCTCAATGAAATAGATATGAATGGTTTTTGCAATCTTTAACACTTTAGATTCTTA
CACTAAAACTTTGTAGACGATCTTAAACTTCCAACAGTTCTTCCATAAAGCTCTAATAGAGATCGCAAACTTTGAAAAACTTGAGTGAT
TCTCAATAAGCTCGAAGGAACCTTCTAGGCTGCTGCTGCGTCGTCCTCCTCGATCTTGGGAGCCAAATAGTACCTTACGTATCCCATAT
CTCCAATCTTATACTCCACCACGATGGGAAGATCGGCAGACATGCTCAGTGTCACGATGTTGGCCAGTGGCGTTGCCTTGGTGAACG
AGTTTAAATAGCGCAGCGCAAACGTGAGCGAGACGGGTTCTTGCATTTTGATTTCCGTGGCGTTCTCGGGCTGGAAAAATAGTGAA
AACCACACAAGCTAGAATCAGTGTGTTGTTTCATTGTAGTTTACCTCGTCCACCGATGTGTTTTGGCGGCACACGATGTTGGCGGTGC
CAATGTCCCCGGAAGTTGTGAACTTGACGCCGTCCTTGGTGACAGAGATCATCACTGGAACGAATAGATACAAACTTTTGATCATAGA
AAACTTTTGAGAGTTCTTTAGTACCTGTGTCGCCAATGCTGCTAAGATCCTTGCAGATGCGCAAGAATTCCTGGGATGGCATCTTGAT
GCTCGATTGGTACTCTGTGTCTGGAATTCCCAGGTGCTCGCTATCGATCTCCATGAGCTTCATCTCAAAGTCAGAGATTTTATCTTGAG
CTGGAAACACAAAGATAGAAAAACTTAGAGCTTGTAGAACAAGAAACGAAGAAACAAGAACTTACAGGGGCTCTCAAACATAAAG
GTCACGGTGTCGCTGCCATCGTCGGCCTTGATGGTGATGATATCGTCGTTTCCAGCGCACTTGAGCATCTTGGCCATATTGGTCAGATT
CATCCCAATGGAAATCGTGCGATCACAGCGATAGTGCTCGAAACCCTCGGACCGGAGCATCAGCGCCACGAGCGCGACATGGCTGG
AATCCATGGCTTGCAGGGAAAATCCAGTGGAAGAGCAATCGAAGTTCGCCTCATTCACGAGATCCTTGATGGCCTCGAGTACCTTCT
TGAGAAGACTTCCCTGGACGAGGCGGAGCTCCAACATCGCGGCCGCGGCGCTGCCAAGAGAGAAGAGCCAAGAAATGCGCGAGT
GATTAAACCCTAAAGCTCGCACCGCAAAAACCCTAGAGCCCCTTTTCACTTTGAAATTTTGGCGGGAACTTAGAGTAGCTTTGGGAT
GCCGTAGCTAAGGCTCGGCAGAGCTTCAATCCTTCTTTATCGTCCATTCATTTTAGGGTTTATAGTTTTCCGTGAGTGGATGGCTGGAA
CATTC

> At-cRPE AT5G61410.1

ATTGTAGTGGAAAATAAAAGATGAAAACTTTGTTGGTCAAAAACTCCTTTTGGTATTATAGGTTTTTTTTTACATTAAGCACGTTTTTG
CTATATATGGGAGCGCTTTATTTTTTTGTCGGCATGTGTGGAGATTTTAATCTAAAGTAATTTGATACTTTTCTTTTAATTTCACTGGTTG
TCAACTTGCAAAAAAAGAGCCCAAGCCCACTCAAATTACGATAATCATATGGCAATATTATTAAGCCAAACCAATACTGTATATAATCCA
ATGTCTTCTTTTGTGACAAAGACACTTTATTTAATGATTAAACGAATACATTATATGCATCATCTAACAAAAAGTAAAAGTGCTGACATAT
CAAAAGACTAGATTCTTATTGCGAGCATTTCATGCTAATAGATCGGTCTTCTTCCGATGGAATCCACTACAATCCATAAAATGTTAATCA
TTGGAGTTCTGTAAACAGAGGATAAGCCACACAAAAAAAACTATATAGATCTGCATATAAATAATTAGGCCTCATGCTAATGGGCTTTA
CTTAAAATAAAGCCTAAAATAGTATTGTGACGAATATGGAGAGACGTTTTGGATTCTCATTCAAATTTCAACCCATTAATGGCTAATCAC
TTTGTAAATGTAAGATTGTACTACTTATATCTTTTACGAGGTGATTTGAATCAATTAAGTAATGCAGAGATTACGATATAATTTTGGGAA
GCACAACTTTTGACCAACACTCCGAATCGGGTCTCTTTCTCTATAAATAGCCATTTCCTAAACACACAATTTTCATACTCTCTCAAACCT

CCAAAATTCTTGATCAATATCAGATAAGAAGAATGAGTGGAAAACGCAGCAATGTCGGTGGAGGAAAGAGCGGTGGCGCTGGAGG
TAAGAGTGGTGGTGGCGGACAAAGCAGTGGCGGAGGAAAAAGCGGCGGCGGAGGAGCAGGAGCAGGTAATATGGTAGCACCAG
GGACAAATGGGGGTGCTTACATCTCAAGAGGTGGATTCGAGAGTAACCCTCAAGGTTACTTTAGTAACTTGCATGGCAGTGGACAA
AGCAAGAAGTGACTTGAGTGTTGTTGTTATACTACAACCATGAGCCATCTTCTTTGATCTATTCTCTATTTAATAAAATGCTATGAAGTA
CAAACAAGTATTGAATAAATGAGATTTTTATTTTTATGAGGTGTTTGTTGCTTTGTGCTTAACCCACTCTTCTTATTTCTTAATCAATCTC
GTTAGTCCTCGTGAACTATGTGCCTAATCCTATGGATCATGGGCCTTCTTTTTTCAGTATGGCCCCATGATATAAAAGCATCATGTAAATA
GATTACGAAATCTATCAAATGGGACTAAATTTGGTATTCTGTTTTTTTCTATAAAATGACCTAAAACATTTTTTGTATATATAATTGGTTGA
CAAAATTATAAAATTTAAAACGATGAGTTTTTCATGTTAATTAAAATTTTCTATCAATTGTTTTAGTCATACATCTCAAATTGAATCTTGAT
TTTAGTATATACATTCCTATTATTTTTTATTTATCAAATATTTTGTAAAAATTATAAATGACCTAAAATTTTTTTTTTTATTAATTAACTTTTTA
TTTAAACGACAATTTATTTAGAAATTATATGTTTTGTATCACTAGTAAAAATAGATTTATTATTTTGCAAAATTAGCATATTCCCACAAATG
TACAAGACCCGTCATCAAAAGAGAGCCAACCGTTTGAGATAGCGATAAGCCGCTGCATTGGATCATCCGAACAAGGAAGACGTTACC
CACGAGACCGTAATCACACGTGGCGGATTCTCAGATATTCTCTCTTTTTCTTCTTTACCCGGAAAAATAAAGTTTCTGTTCTTCTTTTTT
CTTCTCTGGGTTTGAGAATTGGTGTAAGAGAAACGATTGAGGTTTTCTCTTATTATCTAAACCCACCAAGTCTTTCAGGATTTTAGCCA
GAGAAGCTTGAGTCTTTGATTAGGGAC

>Os-cRPE LOC_Os03g07300

GCAGTTATCTATATGTAAATAGTGATAGTCTTGCGGTGAGATCAACTAATTCTAACTACTAGCATAGTTACTTTTGAATATGTACTTTTTAT
GTGCTCTGTTGAAGCATGTGCTTCATCTCATTGCCTCAGCAGCTTCAGCTGCTTTTTGAGATTTGAGAATTATTATTTGTTTGTTTCATG
TTCCATGATTGTTGCTTCTTGAATGTTGCAGGTCTCAAAATACAAGAAGGGTTTGAGGAATGGACCGAAAGCATTGAAGCCGGTTCC
AGTGATTGTCCGTTGCAGGTAAATTTGAAGCTGAAAACAACATGCTTGTTAGTTATTCTGATTTGTTCTGCTATGTCTAAAGACAACTT
AGTATTAGTAAATATCATTTTTTTATGTAACAGCATACTTTATTCTGATTGTTTCACTACTCATGTGGACAATTATTAGACTGTTCAGATTG
TTGTCTAGCGATACCCTCACTCTTTACAATCTAGAATGCAATAGTATCAATAGCCACCAACTTAGGTTTTAGTGGAGCCCAAAGTGCAG
ACACAGAAAACATGCTATGTGATCTAGCATCACAAATGCTGTAGAACAACATTTTGGCGTGCATCAGTTCTTCCCAGATGATTCAGAAA
TTGCGCGACTCATTTCAGTTTCCGGATTTGTCATCCTTTGCTCATTGTTGTTTTTTGTTCCATGAAATTTATTTACATAATTGTTCTGGAG
TACCTTTCCATTGTGCTGACGCTGAAAATTGTTCATCTTGAATGGCATGTTGCAAAGTTTATATAACCTGGTATCCTTTTTGTGCCTGTC
AGGTGCTGTGGCCGAGTGAAGCTGCCCCACTTCTACTGCTGTAGCGGAGAAAAAGGGAACCCCGGCGACTCAAGCTCATAAACCTG
TTCTGTTTCTCCCCAAGTTCAGTCTGCACTCAGCACATCAAGCAATGTCTGAACACTATTCCACCATGAACGTTTTCAGATCTCATTTTG
TGTTCGGCCGCTGTTCTGTTTTCAAGCTTTTTAATCAGTGTACACACTTTAAACCCAGCTTGGATTTCAAAACTCCCCTTGTCAAAAAC
TCAAATGCCACTCGTGACATGAATGGATGCATTCTGTCTGAGACAAGAACCAAATGTGTGCATGTGCCTTGCTAGAAGAAAGAAATA
AAGGGCCTCATCTTTTTTTTGCTTATGCTTATGTTTATCAGCTAAAATTTAAATTTTCAACCTTAAATTTGGAGTTGATTTTGGGGTTTTT
TAATCAAAGTTTATTTTTCAGCCCTTGTTTTTAGATAGCTAAGAACACGTATATAAAACAATTAAAGTTGTATTCACGAATTATTTTTCGT
TTGCCAATATGCCGTTTCGCTTATTCCCTGGTAGAGCCGAAAAGTGTAACAATCCAGCAGCACGGACAGGGGCGAAGCTAGCATCAA
AATAAGAGGGGTGCACTACCATTGAGAATCTAGAAAATTTCAGCACATAACTGCACATTCTAGTATTTTTAAGTCCGAAATTAGGAATC
TGGCACATAGTATAGTTTCGCCACTGCCGCACGGAAGGTGGGCGAGGAAGACTGGAAGAGAGATGCCAGTTCGAGATGCGGATGC
TCTTTCATTTTCGTGCCGTTTCCGGTGCGGTGCAGGTGCAAAAATGCTGCAACCACACGCGCGCAGTGGTGGTGCGGGTTTGGTAG
AGGACGAAACCGCGGTCGTTGGCTTGGCCTTGGCTGGCTCGCCTCAGAGCCGCCACCAACTAACCAACCAAAAACTCCTCCCCAAC
CCAAAAACGCGACGAAATTCGCTGGCCCCCACGCTGCGATTTATGTGGACCGGCTGCGGCTTGGGTGCAGCAGCGAGCAGATAACG
AGGCGAAGCGGAGCCAGATTTCCCTCTCCTCTCCTCTCCTCCTACGCGACGCCTCCTAGCCGCCACCGCTTCACCAAATCAAAAGCTC
TCGCTGCTCCTCGAGAGGGAGTTGGTGGAGAGAGAAAGAGAGAGAGAGAG
