# Supplementary material for: Reduction in chloroplastic ribulose-5-phosphate-3-epimerase decreases photosynthetic capacity in Arabidopsis
Source: Front Plant Sci. 2022 Oct 14;13:813241. doi: 10.3389/fpls.2022.813241 (PMC9614318; doi:10.3389/fpls.2022.813241)
Supplement: Supplementary file 2 [file DataSheet_2.docx]

**
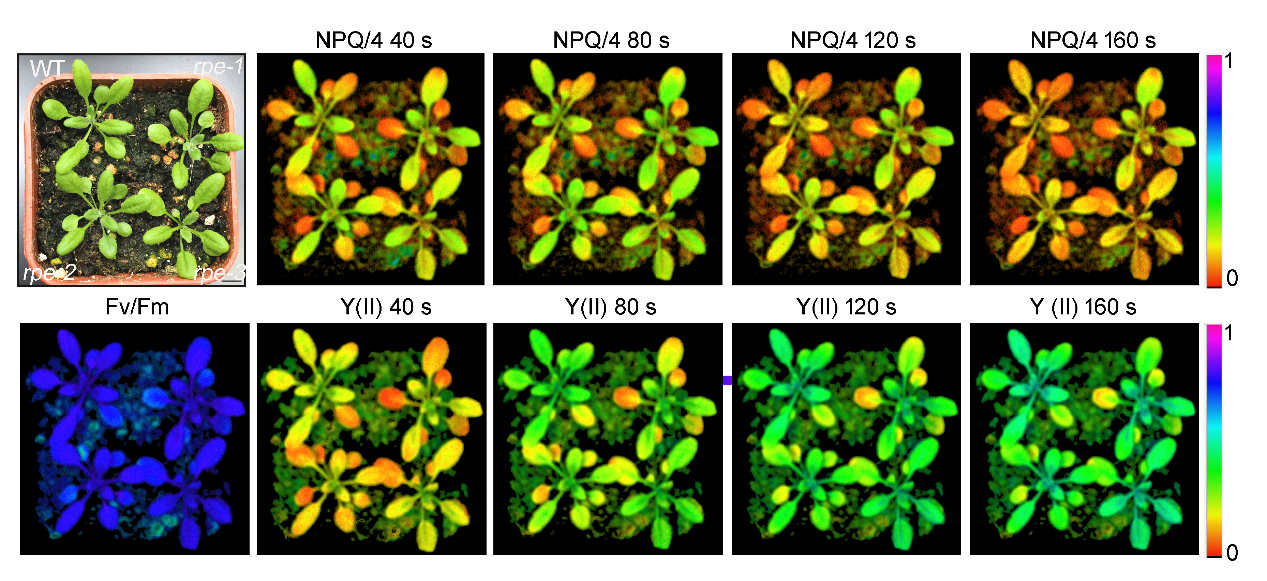
**

**Supplementary Figure 1 |** Growth, NPQ, Fv/Fm and Y(II) phenotypes of WT and *rpe* mutants under growth chamber conditions. Four-week-old plants were measured with default programs using IMAGING-PAM fluorometer. Signal intensities for NPQ, Fv/Fm, and Y(II) are indicated according to the color scale (0 to 1.0) on the right. The bar represents one centimeter.


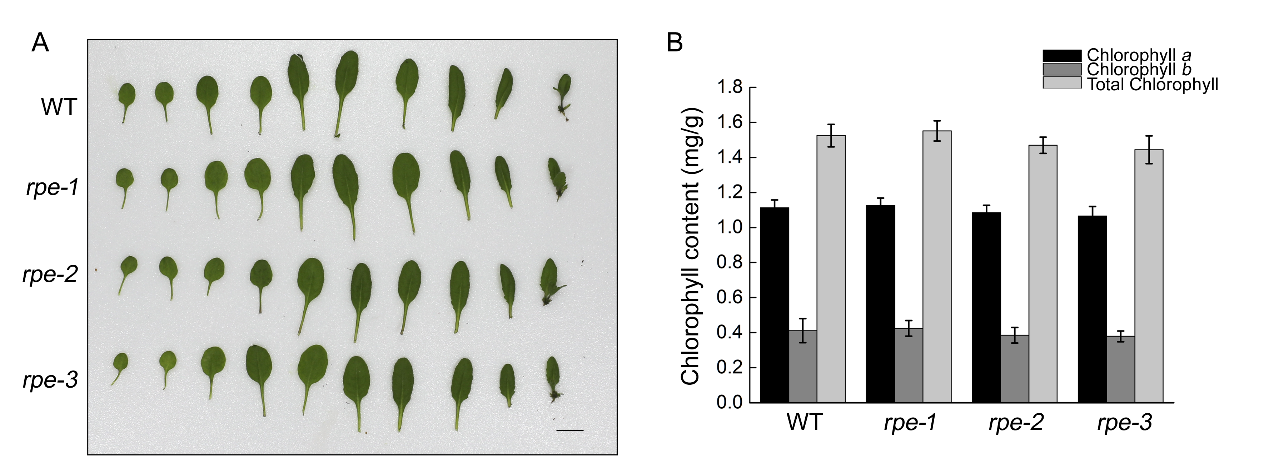


**Supplementary Figure 2** | Leaf morphology and chlorophyll content of WT and *rpe* mutants. **(A)**Leaves of wild-type and mutant plants grown for four weeks were isolated and placed for photographs. The bar represents one centimeter. (B) Fresh and clean leaves of 100 mg were weighed and cut into thin filaments. Put the prepared filaments into tubes (The tubes were wrapped in foil), add 10 mL absolute ethanol, and soak overnight in the dark, shake the filaments until the leaves are colorless or white. Mix the tube thoroughly and measure the absorbance of chlorophyll content using spectrophotometry. The chlorophyll concentrations are calculated as follows. Chlorophyll a: Ca (mg/g) = (12.7D663 nm - 2.69D645 nm)×V/(1000×m)；Chlorophyll b: Cb (mg/g) = (22.9D645 nm - 4.68D663 nm]×V/(1000×m); Chlorophyll a + b: Ca+b (mg/g) = (8.02D663 nm + 20.21D645 nm)×V/(1000×m). V = volume of the extract (mL); m = Weight of fresh leaves (g).


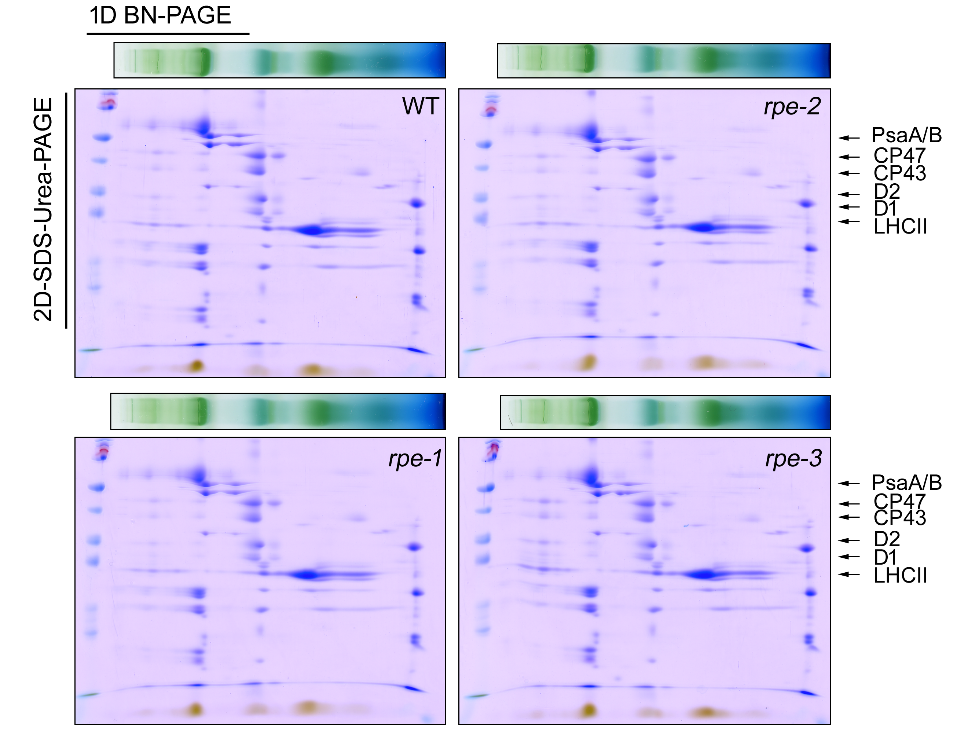


**Supplementary Figure 3 |** 2D BN/ SDS-Urea-PAGE analysis of the thylakoid protein complexes. Thylakoid protein complexes were separated by BN-PAGE, and further resolved by SDS-Urea-PAGE. The gels were stained with CBB.


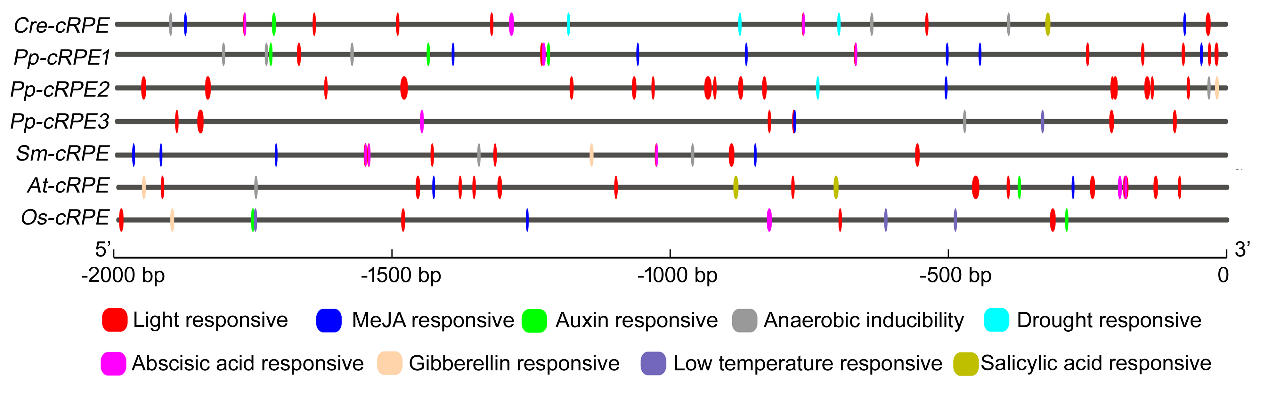


**Supplementary Figure 4 |** Cis-element analysis of the 2 kb upstream region of cRPE genes. *cRPE* represents the *RPE* gene localized in chloroplasts, and there are also *RPE* genes localized in cytoplasm in plants. The 2000 bp upstream region of the candidate *cRPE* genes was extracted from the phytozome website. Cis-regulatory elements (CREs) were detected using online software PlantCARE (Lescot et al., 2002). The figure shows nine cis-elements, one light-responsive regulatory element, five specific phytohormone-related cis-elements (MeJA, auxin, abscisic acid, gibberellin, salicylic acid), and three stress-responsive regulatory elements including drought, low-temperature, and anaerobic. They are illustrated by the different color rounded rectangle boxes. The candidate *cRPE* genes: *Chlamydomonas reinhardtii: Cre12.g511900.t1.2*/*Cre-cRPE*; *Physcomitrella patens:* *Pp3c1_8270V3.1*/*Pp-cRPE1*, *Pp3c7_23810V3.1*/*Pp-cRPE2*, *Pp3c11_3710V3.1*/*Pp-cRPE3*; *Selaginella moellendorffii:* *228435*/*Sm-cRPE*; *Oryza sativa: LOC_Os03g07300.1*/*Os-cRPE*; *Arabidopsis thaliana: AT5G61410.1*/*At-cRPE*.
